# Multiomic Analysis Reveals Disruption of Cholesterol Homeostasis by Cannabidiol in Human Cell Lines

**DOI:** 10.1016/j.mcpro.2022.100262

**Published:** 2022-06-24

**Authors:** Steven E. Guard, Douglas A. Chapnick, Zachary C. Poss, Christopher C. Ebmeier, Jeremy Jacobsen, Travis Nemkov, Kerri A. Ball, Kristofor J. Webb, Helen L. Simpson, Stephen Coleman, Eric Bunker, Adrian Ramirez, Julie A. Reisz, Robert Sievers, Michael H.B. Stowell, Angelo D’Alessandro, Xuedong Liu, William M. Old

**Affiliations:** 1 Department of Molecular, Cellular & Developmental Biology, University of Colorado Boulder, Boulder, Colorado, USA; 2 Department of Biochemistry, University of Colorado Boulder, Boulder, Colorado, USA; 3 Department of Biochemistry and Molecular Genetics, University of Colorado Denver, Aurora, Colorado, USA; 4 Department of Chemistry and Cooperative Institute for Research in Environmental Sciences, University of Colorado Boulder, Boulder, Colorado, USA

**Keywords:** cannabidiol, proteomics, phosphoproteomics, transcriptomics, lipidomics, metabolomics, systems pharmacology, FRET biosensor, high-content screening, multiomics, ACACA, acetyl-CoA carboxylase, ACN, acetonitrile, AGC, automatic gain control, CaMKKβ, Ca^2+^/calmodulin-dependent protein kinase kinase β, CBD, cannabidiol, CFP, cyan fluorescent protein, DAPI, 4′,6-diamidino-2-phenylindole, DARPA, Defense Advanced Research Projects Agency, DHA, docosahexaenoic acid, EEF2, eukaryotic elongation factor 2, ER, endoplasmic reticulum, FA, fatty acid, FASP, filter-aided sample preparation, FDR, false discovery rate, FRAP, fluorescence recovery after photobleaching, GO, Gene Ontology, GRAM, glucosyltransferase, Rab-like GTPase activator, and myotubularin, HEK293T, human embryonic kidney 293T cell line, HK1, hexokinase 1, HMGCR, 3-hydroxy-3-methyl-glutaryl-coenzyme A reductase, IT, injection time, MBCD, methyl-beta-cyclodextrin, MCMC, Markov chain Monte Carlo, MS, mass spectrometry, NMWL, nominal molecular weight limit, NPC1, Niemann–Pick C1, 25-OHC, 25-hydroxycholesterol, rcf, relative centrifugal force, SM, sphingomyelin, SUV, small unilamellar vesicle, TMT, tandem mass tag, TRITC, tetramethylrhodamine, TRPV, transient receptor potential cation channel

## Abstract

The nonpsychoactive cannabinoid, cannabidiol (CBD), is Food and Dug Administration approved for treatment of two drug-resistant epileptic disorders and is seeing increased use among the general public, yet the mechanisms that underlie its therapeutic effects and side-effect profiles remain unclear. Here, we report a systems-level analysis of CBD action in human cell lines using temporal multiomic profiling. FRET-based biosensor screening revealed that CBD elicits a sharp rise in cytosolic calcium, and activation of AMP-activated protein kinase in human keratinocyte and neuroblastoma cell lines. CBD treatment leads to alterations in the abundance of metabolites, mRNA transcripts, and proteins associated with activation of cholesterol biosynthesis, transport, and storage. We found that CBD rapidly incorporates into cellular membranes, alters cholesterol accessibility, and disrupts cholesterol-dependent membrane properties. Sustained treatment with high concentrations of CBD induces apoptosis in a dose-dependent manner. CBD-induced apoptosis is rescued by inhibition of cholesterol synthesis and potentiated by compounds that disrupt cholesterol trafficking and storage. Our data point to a pharmacological interaction of CBD with cholesterol homeostasis pathways, with potential implications in its therapeutic use.

## Highlights

• Cannabidiol (CBD) treatment elicits a broad spectrum of intracellular phenotypes.
• Cytosolic and ER calcium homeostasis is disturbed by CBD.
• CBD incorporates into membranes and alters cholesterol accessibility.
• Cholesterol biosynthesis and transport signaling is increased by CBD.
• Lateral diffusion of cholesterol within membranes is decreased by CBD.

## Graphical Abstract

**Figure:**
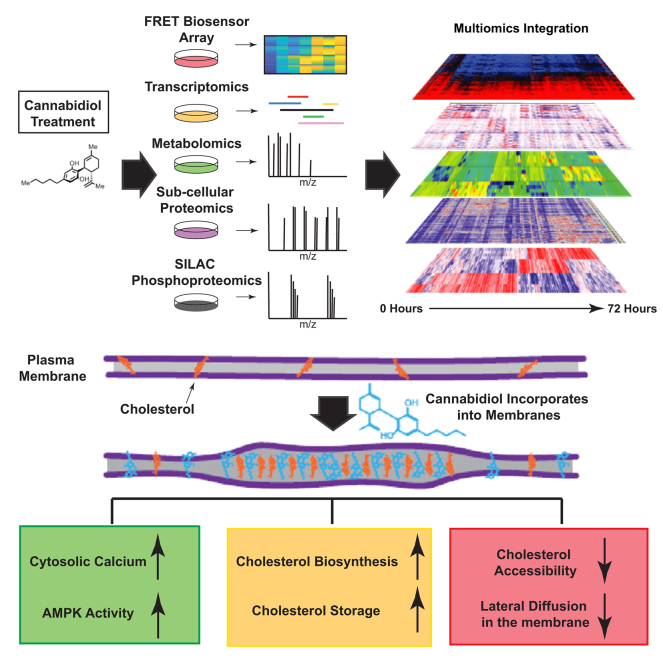

## In Brief

The nonpsychoactive cannabinoid, cannabidiol (CBD) has been Food and Drug Administration approved for treatment of two drug-resistant epileptic disorders and is seeing widespread supplemental use by the public. The mechanisms that underly the therapeutic effects of CBD are not well understood. We took a systematic multiomics approach to profile the intracellular pathway changes that occur following CBD treatment. From this effort, we identified a response centered in the disruption of cholesterol homeostasis and membrane fluidity that could impact clinical outcomes.

The nonpsychoactive cannabinoid, cannabidiol (CBD), was recently approved by the Food and Drug Adminstration for reducing convulsive seizure frequency in the drug-resistant epileptic disorders, Lennox–Gastaut syndrome, and Dravet syndrome (1, 2, 3). CBD lacks the psychotropic effects of its structural isomer, tetrahydrocannabinol, because of weak binding affinity (>3–10 μM) for the endocannabinoid receptor CB_1_ (4). CBD is well tolerated in humans (5) and has been proposed as a potential therapeutic for a wide range of conditions, including colitis (6), cancer (7, 8, 9, 10), neuroinflammation (11), chemotherapy-associated nephrotoxicity (12), cardiomyopathy, and diabetic complications (13). Despite decades of research, the molecular mechanisms that underlie its therapeutic effects and side-effect profile are not well understood (1, 3, 14, 15).

More than 65 protein targets of CBD have been proposed, 22 of which are membrane-localized channels and receptors (16, 17, 18, 19). For example, CBD has been shown to inhibit voltage-dependent sodium currents mediated by the NaV1.1 sodium channel (20), which is mutated in Dravet syndrome (21). CBD has also been shown to inhibit voltage-dependent ion currents of six other human sodium channels, the Kv2.1 potassium channel, and even a bacterial sodium channel, with IC_50_ values of 1 to 3 μM (20). Proposed targets also include calcium channels or receptors that regulate calcium, including T-type calcium channels (22), voltage-dependent anion channel 1 (23), G protein–coupled receptor 55 (18), voltage-gated calcium channel Cav3.x (22), and transient receptor potential cation channels 1 to 4 (TRPV1–4) (17). Postsynaptic calcium mobilization has been proposed as a mechanism to explain the anticonvulsant activity of CBD (24). The ability of CBD to modulate many structurally diverse membrane channels and receptors raises the question of whether it acts through nonspecific mechanisms, for example, through biophysical alteration of lipid bilayers in which many of the proposed targets reside (20, 25, 26, 27).

Comparatively less is known about intracellular targets and pathways engaged by CBD in humans. In microglial cells, CBD has anti-inflammatory activity and upregulates mRNA transcripts involved in fatty acid (FA) metabolism and cholesterol biosynthesis (28). In adipocytes, CBD leads to a reduction in triglyceride levels, concomitant with phosphorylation changes of regulatory proteins controlling lipid metabolism, including cAMP-response element binding protein (CREB), AMP-activated protein kinase A2 (AMPKA2), and heat shock protein 60 (HSP60) (29). In mice, CBD attenuates liver steatosis and metabolic dysregulation associated with chronic alcohol feeding (30). These studies point to a systematic modulation of lipid and cholesterol pathways by CBD in animal models and human cell lines *via* yet unknown mechanisms. The large-scale alteration in transcripts, proteins, and metabolites across numerous pathways suggests that CBD acts pleiotropically through numerous biomolecular targets and/or nonspecific effects on cellular membranes. This evidence motivated us to use an unbiased systems-based approach to examine the molecular basis of CBD cellular perturbation.

Recent advances in mass spectrometry (MS) have enabled comprehensive identification and quantification of cellular proteomes and metabolomes (31). Multiomic profiling strategies that combine MS-based proteomics with transcriptome profiling can reveal critical and unexpected insights into the mechanisms of drug action (32, 33). In this study, we examined phenotypic and molecular responses to CBD treatment in a human neuroblastoma cell line, SK-N-BE(2), using four complementary experimental approaches: (1) high-content imaging of FRET biosensors monitoring a panel of cellular activities, (2) subcellular proteomics, (3) phosphoproteomics, and (4) flux metabolomics. We found that CBD led to a chronic rise in cytosolic calcium and activation of 5′-AMPK signaling within 3 h post-treatment. In SK-N-BE(2) cells grown in cholesterol-replete media, CBD treatment led to increased abundance of mRNA transcripts and proteins involved in cholesterol import and biosynthesis. Metabolomics revealed a concomitant CBD-dependent increase in flux of glucose-derived carbon through cholesterol biosynthesis intermediates, despite being grown in cholesterol-replete conditions, suggesting that cholesterol sensing and synthesis become decoupled in the presence of CBD. We further show that CBD sensitizes human cells to apoptosis when cotreated with inhibitors of cholesterol trafficking and storage. Conversely, atorvastatin, an inhibitor of cholesterol biosynthesis, rescued cells from CBD-induced apoptosis. Together, our data reveal that CBD partitions into cellular membranes and leads to disruption of cholesterol homeostasis and membrane-dependent processes.

## Experimental Procedures

### Compound Preparation

CBD was derived from domestically grown industrial hemp that was cultivated and purified by Sievers Infinity, LLC, Colorado-owned corporation, registered with the Colorado Department of Agriculture to grow and cultivate industrial hemp (Colorado Department of Agriculture; 69096). The purified hemp-derived material was characterized by MS, X-ray diffraction, differential scanning calorimetry, NMR (1H-NMR and 13C-NMR) spectroscopy, and HPLC-UV. The quantitative proton NMR results indicate that the sample is >95% CBD, and the HPLC results indicate that 12 other commonly found cannabinoids (including delta-9-tetrahydrocannabinol) were less than the limit of detection of 0.004%.

### Generation of FRET Biosensor Cell Lines

Stable transgenic biosensor-expressing cell lines were made in HaCaT and SK-N-BE(2) cells as previously described (34). Briefly, biosensor gene–containing plasmids were obtained through the Addgene plasmid depository and subcloned into our Bsr2 parent plasmid (sequence available upon request). Each biosensor Bsr2 plasmid was cotransfected with a PB recombinase–expressing vector (mPB) *via* polymer-based transfection using polyethyleneimine (Polysciences; 25 kD linear). Each stable transgenic cell line was selected for 7 days using 10 μg/ml blasticidin S. FRET biosensor profiling was conducted in multiplexed parallel live-cell experiments using 384-well imaging plates (Corning; catalog no.: 3985) in an ImageXpress MicroXL high-throughput microscope. Filters used for FRET measurements were the following: FRET excitation 438/24-25, dichroic 520LP, emission 542/27-25 (Semrock; catalog no.: MOLE-0189); cyan fluorescent protein (CFP) excitation 438/24-25, dichroic 458LP, emission 483/32-25 (Semrock; catalog no.: CFP-2432B-NTE-Zero). Time-lapse microscopy images were collected, and FRET ratio calculations for each site in each well at each time were performed as the mean value from pixels above threshold of background and flat-field corrected images, where each pixel value represented the FRET channel intensity divided by the CFP channel intensity. This method is described in more detail in our previous studies (34, 35). Calculation and data visualization were performed in MATLAB (MathWorks) using custom scripts that are available upon request.

### EC_50_ Estimation From FRET Sensor Dose Responses

Dose responses at each time point were fit with the following fit function *y* = 1/(1 + np.exp[−k ∗ (*x* − EC_50_)]) using python's scipy.optimize.curve_fit package. Prior to fitting, measurements were scaled between zero and one. *R*^2^ goodness of fit (GOF) was calculated between the sigmoid fit and the median of the replicates (duplicates) for each sensor/time point combination. Fits with EC_50_ estimates outside the dose range were discarded. EC_50_ values were kept for fits that resulted in an *R*^2^ goodness of fit >0.75. The resulting distribution of EC_50_ values was somewhat bimodal resulting in a median EC_50_ of 8.48 μM across all sensors and times.

### Transcriptomics Workflow

CBD and vehicle treatments were prepared in quadruplicate (four drug treated/four vehicle controls) at 3, 6, 12, and 24 h time points. About 500 ng of total RNA was used in Illumina TruSEQ mRNA library prep protocol. Libraries were run on the Illumina HiSEQ4000 at single-read 50 bp length. Sequencing was performed on seven consecutive lanes. Median read counts per lane were ∼49,000 with a CV of ∼7%. Starting with 228 fastq files, each lane set was concatenated per condition. Run specifications were 51 bp reads, standard single read, and first stranded. Alignment to the human genome (HG19) was done using Tophat (v2.0.1.3). Two mismatches were allowed, and duplicate mapping was permitted at up to two locations. Using Trimmomatic (v0.36), base pairs were trimmed if they dropped below a PHRED score of 15 within a sliding window of 4 bp. Remaining reads shorter than 35 base pairs were removed. Illumina adapter sequences were also clipped using Trimmomatic. Fastqc was used to verify data integrity before and after trimming. Cufflinks/Cuffdiff (v2.2.1) was used to obtain fragments per kilobase of exon per million mapped reads' normalized gene count and differential expression measurements at each time point (36). One *p*/*q* value was generated for each gene at each time point. Genes with a *q* value significance of <0.05, and absolute log_2_ fold change of 1 or greater, for at least one time point, were retained for downstream analysis.

### Proteomics

#### Experimental Design and Statistical Rationale

Protein quantification for time series was performed with a tandem mass tag (TMT) isobarically labeled 11-plex multiplexing scheme. The 15-point time series for each cellular fraction was split into three series, with every series containing five treatment and matched control time point pairs, with 0 s, 40 min, 3 h, 12 h, and 24 h time points in series A; 10 min, 80 min, 6 h, 15 h, and 48 h in series B; and 20 min, 2 h, 9 h, 18 h, and 72 h time points in series C. This separation was performed so that a protein could be missing from one and/or two series because of stochastic effects in data-dependent acquisition, and the overall trend could still be inferred, though with reduced resolution. The 11th label in each series was devoted to a global mix reference channel, which would be present in all series for a given cellular fraction. The global mix is a cell fraction–specific mixture that contains an equal portion from each time point sample. This channel was the denominator in the intermediate ratiometric measurement for differential expression for both drug-treated samples and time-matched controls. This mixture channel was constructed so that every measurable protein observed at any time point has a nonzero denominator when ratios are taken. When the differential expression is compared between the drug-treated labeled samples and matched control samples and expressed as a log_2_ ratio, the global mix reference channel cancels out.

The differential expression of each individual protein was determined using Bayesian methods for isobaric-labeled proteomics data (37). Briefly, all observed peptides are mapped to a list of observed protein IDs *via* Isoform Resolver (Natalie Ahn's Research Group) (38). The TMT 11-plex reporter ion spectrum peaks for each peptide contributes to the inference of the differential expression of a protein and reporter ion label. In this case, each reporter ion label represents a measured time point. The label-to-label normalization is handled *via* a hierarchical model, which calculates the bias inherent with each specific label by pooling differential expression estimates from all proteins, changing and unchanging. The hierarchical models are solved computationally with a Markov chain Monte Carlo (MCMC) method, running chains in parallel for faster results (39). The MCMC returns a Gaussian posterior probability distribution of log_2_ differential expression for each protein for each label. The model initially fits the ratiometric differential expression for every treatment and matched control relative to a global mix channel, and the reported drug-induced differential expression is the difference (in log_2_ space) between the treated sample and the matched control sample. Five MCMC chains were run independently for at least 500 k steps, and convergences for each run were verified *via* Gelman–Rubin convergence variable <1.05 (40).

The differential expression was calculated independently for all biological replicates so protein-level variance from separate replicates could be examined and quantified in the posterior distributions obtained from MCMC. For reporting a single differential expression for a protein and label, the Bayesian updating procedure is used to produce a single posterior distribution, from which a mean point estimate and 95% credible interval are calculated. In some specific instances, labels represent technical rather than biological replicates. In cases of technical replicates, the point estimate values were averaged, and the credible interval extents were treated as errors and added in quadrature. With this procedure, technical replicates contribute a single probability distribution to any further Bayesian updating.

For every cellular fraction and time point, then, there are between three and six biological replicates, and the number of replicates represented in the drug-treated samples and the matched control samples are not necessarily the same. The effect size (Cohen’s *d*) was calculated between the posterior probability distributions of the drug-treated and matched control samples as a standardized measure to determine if there was a drug effect. Statistical power analysis was performed to show that, with significance criteria α = 0.05 and the statistical power requirement (1 − β) = 0.8, the appropriate effect size threshold should be *d* > (1.50, 1.75, 2.25, 3.38) for proteins observed within 6, 5, 4, or 3 replicates, respectively. A protein was selected for further consideration if it showed differential expression greater than this threshold for any given time point.

The Bioconductor Edge package (41) (https://doi.org/10.18129/B9.bioc.edge), version 2.8.0, was used for time-course differential analysis. Many proteins were not present for all replicates and/or plexes, so Edge was run sequentially to generate *p* values for each case. For instance, in the soluble fraction, there were 273 proteins that were only present in two replicates. These were run through Edge separately from the other 1957 proteins that were observed in three replicates. The resulting time series *p* values were combined into a list and false discovery rate (FDR) corrected using Benjamini–Hochberg multiple hypothesis correction (42).

### Proteomics Network Analysis

Of the significantly changing proteins, correlation networks were generated for each subcellular fraction. Networks were created from the ethanol- (vehicle) treated samples as well as for the CBD-treated samples. Network edge values were assigned using Spearman correlation coefficients between all proteins (vertices) for a given replicate. For each pair of proteins, 2∗N edge values were generated, where N is the number of available replicate measurements for that protein. An independent *t* test was used between basal replicate edge values and treatment edge values to evaluate what edges were significantly changed because of CBD treatment. Edges with −log_10_(*p* value) >2 (*p* < 1%) were retained. Python graph-tool package was used to generate a stochastic block-model representation of the resulting network, which clusters nodes based on network connectivity similarity.

#### Combined Heatmap Criteria

All protein IDs with edge-adjusted *p* values less than 1% were merged with gene IDs from RNA-Seq with edge-adjusted *p* value <1% and minimum absolute log_2_ fold change >0.5. Merged list was used as input for Enrichr (Ma'ayan Laboratory) (43) to get a table of Gene Ontology (GO) terms (go_biological_processes_2017). GO terms were reduced using REVIGO with “medium” size setting: Terms with dispensability score less than 0.1 and *q* value <5% were kept. Merged IDs from remaining GO ontologies were clustered and plotted in heatmap by relative expression in CBD-treated condition compared with vehicle control at each time point starting at 3 h.

### Subcellular Fractionation in Proteomics

For each sample, a 10 cm petri dish containing 10^6^ SK-N-BE(2) cells was harvested and washed three times with 10 ml of 20 °C PBS. All PBSs were removed by aspiration, and plates were frozen using liquid nitrogen and stored at −80 °C overnight. Each plate was thawed on ice and 400 μl Tween-20 buffer (1× PBS, 0.1% Tween-20, 5 mM EDTA, 30 mM NaF, 1 mM NaVo_4_, 100 μM leupeptin, and 2.5 μM pepstatin A) and scraped thoroughly using a standard cell scraper. The resulting lysate was homogenized with a 200 μl pipette and transferred to 1.7 ml Eppendorf tube on ice. Lysate tubes were incubated for 30 min at 4 °C rotating end over end. After rotation, tubes were centrifuged for 10 min at 4 °C (16,100 relative centrifugal force [rcf]). All supernatants were transferred into new labeled 1.7 ml Eppendorf. This tube contains insoluble buoyant plasma membrane and cytosol. The leftover pellet is the “membrane” fraction and is enriched in nuclei. About 40 μl of 1 M NaOAc was added to the supernatants, which immediately were exposed to centrifugation for 10 min at 4 °C (16,100 rcf). All supernatants were transferred into new labeled 1.7 ml Eppendorf. This is the “soluble” fraction. The pellet was resuspended in 400 μl 20 °C SDS buffer. This is “insoluble #2” fraction. All fraction-containing tubes were filled completely with −20 °C acetone and stored overnight in −20 °C. Each tube was exposed to centrifugation for 10 min at 4 °C (16,100 rcf), and supernatants were aspirated and discarded, whereas pellets were allowed to air dry for 10 min at 20 °C. The pellets then proceeded to the filter-aided sample preparation (FASP) procedure.

### Quantitative Subcellular Proteomics

#### Sample Preparation

Precipitated and dried subcellular protein extracts were solubilized with 4% (w/v) SDS, 10 mM Tris(2-carboxyethyl)phosphine, and 40 mM chloroacetamide with 100 mM Tris base (pH 8.5). SDS lysates were boiled at 95 °C for 10 min and then 10 cycles in a Bioruptor Pico (Diagenode) of 30 s on and 30 s off per cycle, or until protein pellets were completely dissolved. Samples were then cleared at 21,130*g* for 10 min at 20 °C and then digested into tryptic peptides using the FASP method (44). Briefly, SDS lysate samples were diluted 10-fold with 8 M urea, 0.1 M Tris (pH 8.5), and loaded onto an Amicon Ultra 0.5 ml 30 kD nominal molecular weight limit (NMWL) cutoff (Millipore) ultrafiltration device. Samples were washed in the filters three times with 8 M urea, 0.1 M Tris (pH 8.5) and again three times with 0.1 M Tris (pH 8.5). Endoproteinase Lys-C (Wako) was added and incubated 2 h rocking at room temperature, followed by trypsin (Pierce), which was incubated overnight rocking at room temperature. Tryptic peptides were eluted *via* centrifugation for 10 min at 10,000*g* and desalted using an Oasis HLB cartridge (Waters) according to the manufacturer’s instructions.

#### High pH C18 Fractionation of TMT-Labeled Peptides

Dried 10-plexed samples were then suspended in 20 μl 3% (v/v) acetonitrile (ACN) and 0.1% (v/v) TFA and loaded onto a custom-fabricated reverse-phase C18 column (0.5 × 200 mm C18, 1.8 μm 120 Å UChrom (nanoLCMS Solutions) maintained at 25 °C and running 15 μl/min with buffer A, 10 mM ammonium formate, pH 10 and buffer B, 10 mM ammonium formate, pH 10, in 80% (v/v) ACN with a Waters M-class UPLC (Waters). Peptides were separated by gradient elution from 3% B to 50% B in 25 min and then from 50% B to 100% B in 5 min. Fractions were collected in seven rounds of concatenation for 30 s per fraction and then combined for a final of six high pH C18 fractions. Samples were dried and stored at −80 °C until ready for LC–MS analyses.

#### LC–MS Analysis

Samples were suspended in 3% (v/v) ACN, 0.1% (v/v) TFA, and directly injected onto a 1.7 μm, 130 Å C18, 75 μm × 250 mm M-class column (Waters), with a Waters M-class UPLC or a nanoLC1000 (Thermo Fisher Scientific). Tryptic peptides were gradient eluted at 300 nl/min, from 3% ACN to 20% ACN in 100 min into an Orbitrap Fusion mass spectrometer (Thermo Fisher Scientific). Precursor mass spectra (MS1) were acquired at 120,000 resolution from 380 to 1500 *m/z* with an automatic gain control (AGC) target of 2 × 10^5^ and a maximum injection time (IT) of 50 ms. Dynamics exclusion was set for 15 s with a mass tolerance of ±10 ppm. Quadrupole isolation for MS2 scans was 1.6 Da sequencing the most intense ions using top speed for a 3 s cycle time. All MS2 sequencings were performed using collision-induced dissociation at 35% collision energy and scanned in the linear ion trap. An AGC target of 1 × 10^4^ and 35 s maximum IT was used. Selected-precursor selections of MS2 scans were used to isolate the five most intense MS2 fragment ions per scan to fragment at 65% collision energy using higher-energy collision dissociation with liberated TMT reporter ions scanned in the Orbitrap at 60,000 resolution (full width at half maximum). An AGC target of 1 × 10^5^ and 240 s maximum IT was used for all MS3 scans. All raw files were converted to mzML files and searched against the UniProt Human database (downloaded April 1, 2015) using Mascot, version 2.5 (MatrixScience), with cysteine carbamidomethylation as a fixed modification. Methionine oxidation and protein N-terminal acetylation were searched as variable modifications. Specificity of proteases: trypsin/P and missed and/or nonspecific cleavages permitted: 2. Peptide mass tolerance was 20 ppm for MS1 and 0.5 mDa for MS2. All peptides were thresholded at a 1% FDR.

### Phosphoproteomics

#### Sample Preparation and Phosphopeptide Enrichment

SK-N-BE(2) cells were cultured in stable isotope labeling of amino acids in cell culture media either with ^13^C_6_^15^N_2_-lysine/^13^C_6_^15^N_4_-arginine (Lys8/Arg10) (heavy) or Lys0 and Arg0 (light). Two biological replicates of near confluent heavy cells and two replicates of near confluent light cells were treated with 20 μM CBD for 10 min (four replicates), 1 h (four replicates), and 3 h (four replicates) for phosphoproteomics analysis. Cells were harvested in 4% (w/v) SDS, 100 mM Tris, pH 8.5, and boiled at 95 °C for 5 min. Samples were reduced with 10 mM Tris(2-carboxyethyl)phosphine and alkylated with 50 mM chloroacetamide and then digested using the FASP protocol, with the following modifications: an Amicon Ultra 0.5 ml 10 kD NMWL cutoff ultrafiltration device was used rather than a 30 kD NMWL cutoff. Tryptic peptides were cleaned by a Water HLB Oasis cartridge (Waters) and eluted with 65% (v/v) can and 1% TFA. Glutamic acid was added to 140 mM and TiO_2_ (Titansphere; GL Sciences) was added at a ratio of 10 mg TiO_2_:1 mg tryptic peptide and incubated for 15 min at an ambient atmosphere. The phosphopeptide-bound TiO_2_ beads were washed with 65% (v/v) ACN, 0.5% TFA, and again with 65% (v/v) ACN, 0.1% TFA, and then transferred to a 200 μl C8 Stage Tip (Thermo Fisher Scientific). Phosphopeptides were eluted with 65% (v/v) ACN, 1% (v/v) ammonium hydroxide, and lyophilized dry.

#### High pH C18 Fractionation of Enriched Phosphopeptides

Enriched phosphopeptide samples were then suspended in 20 μl 3% (v/v) ACN and 0.1% (v/v) TFA and loaded onto a custom-fabricated reverse-phase C18 column (0.5 × 200 mm C18, 1.8 μm 120 Å UChrom maintained at 25 °C and running 15 μl/min with buffer A, 10 mM ammonium formate, pH 10 and buffer B, 10 mM ammonium formate, pH 10 in 80% [v/v] ACN with a Waters M-class UPLC [Waters]). Peptides were separated by gradient elution from 3% B to 50% B in 25 min and then from 50% B to 100% B in 5 min. Fractions were collected in seven rounds of concatenation for 30 s per fraction for a final of 12 high pH C18 fractions. Samples were dried and stored at −80 °C until analysis.

#### LC–MS Analysis of Phosphopeptide Fractions

Samples were suspended in 3% (v/v) ACN, 0.1% (v/v) TFA, and directly injected onto a 1.7 μm, 130 Å C18, 75 μm × 250 mm M-class column, with a Waters M-class UPLC. Tryptic peptides were gradient eluted at 300 nl/min, from 3% ACN to 20% ACN in 100 min into an Orbitrap Fusion mass spectrometer. Precursor mass spectrums (MS1) were acquired at 120,000 resolution from 380 to 1500 *m/z* with an AGC target of 2 × 10^5^ and a maximum IT of 50 ms. Dynamic exclusion was set to 20 s with a mass tolerance of ±10 ppm. Isolation for MS2 scans was 1.6 Da using the quadrupole, and the most intense ions were sequenced using top speed for a 3 s cycle time. All MS2 sequencings were performed using higher-energy collision dissociation at 35% collision energy and scanned in the linear ion trap. An AGC target of 1 × 10^4^ and 35 s maximum IT was used. Raw files were searched against the UniProt human database (downloaded April 1, 2015) using MaxQuant (v1.6.0.13) with cysteine carbamidomethylation as a fixed modification. Specificity of proteases: trypsin/P; missed and/or nonspecific cleavages permitted: 2; and a mass tolerance of 20 ppm for MS1 and 0.5 Da for MS2. Methionine oxidation, protein N-terminal acetylation, and phosphorylation of serine, threonine, and tyrosine were searched as variable modifications. All peptides and proteins were thresholded at a 1% FDR.

#### Bulk Metabolomics Sample Preparation

Cultured cells were harvested, washed with PBS, flash frozen, and stored at −80 °C until analysis. Prior to LC–MS analysis, samples were placed on ice and resuspended with methanol:ACN:water (5:3:2, v/v/v) at a concentration of 2 million cells per ml. Suspensions were vortexed continuously for 30 min at 4 °C. Insoluble material was removed by centrifugation at 10,000*g* for 10 min at 4 °C, and supernatants were isolated for metabolomics analysis by UHPLC–MS. This method was used for cholesterol precursors and free head groups.

#### UHPLC–MS Analysis for Bulk Metabolomics

Analyses were performed as previously published (45, 46). Briefly, the analytical platform employs a Vanquish UHPLC system (Thermo Fisher Scientific) coupled online to a Q Exactive mass spectrometer (Thermo Fisher Scientific). Samples were resolved over a Kinetex C18 column, 2.1 × 150 mm, 1.7 μm particle size (Phenomenex) equipped with a guard column (SecurityGuard ULTRA cartridge—UHPLC C18 for 2.1 mm ID columns—AJO-8782; Phenomenex) (A) of water and 0.1% formic acid and a mobile phase (B) of ACN and 0.1% formic acid for positive ion polarity mode, and an aqueous phase (A) of water:ACN (95:5) with 1 mM ammonium acetate and a mobile phase (B) of ACN:water (95:5) with 1 mM ammonium acetate for negative ion polarity mode. Samples were eluted from the column using either an isocratic elution of 5% B flowed at 250 μl/min and 25 ºC or a gradient from 5% to 95% B over 1 min, followed by an isocratic hold at 95% B for 2 min, flowed at 400 μl/min and 30 ºC. The Q Exactive mass spectrometer was operated independently in positive or negative ion mode, scanning in full MS mode (2 μscans) from 60 to 900 *m/z* at 70,000 resolution, with 4 kV spray voltage, 15 sheath gas, and 5 auxiliary gas. Calibration was performed prior to analysis using the Pierce Positive and Negative Ion Calibration Solutions (Thermo Fisher Scientific). Acquired data were then converted from raw to .mzXML file format using Mass Matrix. Metabolite assignments, isotopologue distributions, and correction for expected natural abundances of deuterium, ^13^C, and ^15^N isotopes were performed using MAVEN (47). Graphs, heatmaps, statistical analyses (either *t* test or ANOVA), metabolic pathway analysis, partial least squares-discriminant analysis, and hierarchical clustering were performed using the MetaboAnalyst package (www.metaboanalyst.com/) (48).

#### Lipidomics Sample Preparation

Extraction of cholesterol, precursors, free FAs, cholesteryl esters, and phospholipids were performed in the following manner. SK-N-BE(2) cells in 10 cm dishes were washed with 10 ml PBS twice and then cells were scraped and pelleted at 400 rcf for 2 min. Cell pellets were resuspended in 100% methanol at 4 °C and sonicated at 70% power in 10 pulses, 5 s on/5 s off. The resulting lysate was rotated for 60 min at room temperature, followed by centrifugation for 20 min at 4 °C (16,100 rcf). Subcellular fractionation of organelles from intact SK-N-BE(2) cells was done in the following manner to assess subcellular CBD distribution. Cells in 10 cm culture dishes were harvested by washing twice with 10 ml PBS at room temperature, followed by trypsinization using a cell culture grade trypsin/EDTA solution (Thermo Fisher Scientific). Trypsinized cells were quenched by addition of 2 ml 10% fetal bovine serum containing Dulbecco's modified Eagle's medium, and cells were pelleted by centrifugation for 2 min at 4 °C (200 rcf). Cell pellets were washed one time with 10 ml PBS and resuspended in 1 ml Tween-20 buffer (1× PBS, 0.05% Tween-20, and 5 mM EDTA). This lysate was subjected to mechanical disruption using a 1 ml glass Dounce homogenizer, 10 full passes at 4 °C. Nuclei were pelleted from homogenate by centrifugation for 5 min at 4 °C (2000 rcf). Supernatant was separated, and insoluble endoplasmic reticulum (ER) membranes were pelleted by centrifugation for 10 min at 4 °C (4000 rcf). Supernatant was separated, and insoluble plasma membranes were pelleted by centrifugation for 10 min at 4 °C (16,000 rcf). Extraction of all fractions was done in 100% methanol for 2 h at room temperature and rotation end over end, followed by removal of insoluble material by centrifugation for 20 min at 20 °C (16,100 rcf).

#### UHPLC–MS Analysis for Lipidomics

Samples were analyzed as published (49). Briefly, analytes were resolved over an ACQUITY HSS T3 column (2.1 × 150 mm, 1.8 μm particle size using an aqueous phase (A) of 25% ACN and 5 mM ammonium acetate and a mobile phase (B) of 90% isopropanol, 10% ACN, and 5 mM ammonium acetate. The column was equilibrated at 30% B, and upon injection of 10 μl of extract, samples were eluted from the column using the solvent gradient: 0 to 9 min 30 to 100% B and 0.325 ml/min; hold at 100% B for 3 min at 0.3 ml/min, and then decrease to 30% over 0.5 min at 0.4 ml/min, followed by a re-equilibration hold at 30% B for 2.5 min at 0.4 ml/min. The Q Exactive mass spectrometer was operated in positive and negative ion modes using electrospray ionization, scanning in full MS mode (2 μscans) from 150 to 1500 *m/z* at 70,000 resolution, with 4 kV spray voltage, 45 shealth gas, and 15 auxiliary gas. When required, data-dependent MS2 was performed at 17,500 resolution, AGC target = 1e5, maximum IT = 50 ms, and stepped normalized collision energy of 25, 35 for positive mode, and 20, 24, and 28 for negative mode. Calibration was performed prior to analysis using the Pierce Positive and Negative Ion Calibration Solutions. Acquired data were then converted from .raw to .mzXML file format using Mass Matrix. Samples were analyzed in randomized order with a technical mixture injected incrementally to qualify instrument performance. This technical mixture was also injected three times per polarity mode and analyzed with the aforementioned parameters, except collision-induced dissociation fragmentation was included for unknown compound identification. Metabolite assignments were made based on accurate intact mass (sub 5 ppm), isotope distributions, and relative retention times, and comparison to analytical standards in the SPLASH Lipidomix Mass Spec Standard (Avanti Polar Lipids) using MAVEN. Discovery mode analysis was performed with standard workflows using Compound Discoverer and Lipid Search 4.0 (Thermo Fisher Scientific).

#### Confocal Microscopy of Cholesterol and Lysosomes

SK-N-BE(2) cells were seeded into fibronectin-coated glass bottom 96-well plates (MatriPlate) at a cell density of 40,000 cells/well using low-background imaging media (FluoroBrite Dulbecco's modified Eagle's medium with all supplements described previously). At the time of seeding, LysoTracker Deep Red (Thermo Fisher Scientific) was added at a 1000× dilution, and NBD-cholesterol (Thermo Fisher Scientific) was added at a final concentration of 10 μg/ml. After 24 h, CBD or ethanol vehicle was added to a final concentration of 20 μM and incubated for an additional 24 h prior to imaging using a Nikon A1R laser scanning confocal microscope for acquisition with the FITC and tetramethylrhodamine (TRITC) channels. In experiments using U18666A, a final concentration of 10 μg/ml was used and added simultaneously with CBD.

#### Assaying Cell Viability and Apoptosis

Cell viability for SK-N-BE(2) cells was conducted using a fluorometric cell viability assay using Resazurin (PromoKine) according to the manufacturer’s instructions. Measurement of percent apoptotic cells was done in 384-well imaging plates (Corning; catalog no.: 3985) seeded with 2000 cells/well and stained with Hoescht 33258 (1 μg/ml) and CellEvent Caspase-3/7 Green Detection Reagent (Thermo Fisher Scientific) at a dilution of 1000×. Dyes were added at the time of seeding, 18 to 24 h prior to performing experiments. For experiments using atorvastatin, atorvastatin was added 24 h prior to addition of CBD. For experiments involving 25-hydroxy cholesterol, U18666A, and VULM 1457, inhibitors were added simultaneously with CBD. Experiments were performed using an ImageXpress MicroXL microscope and a 10× objective, where images were acquired for each well at the indicated time points using 4′,6-diamidino-2-phenylindole (DAPI) and FITC filter sets. Using MATLAB, images were processed with custom written scripts (available upon request) that perform flat-field and background correction, identification of all cells (DAPI channel) using standard threshold above background techniques, and identification of apoptotic cells using a similar method in the FITC channel. Percent of apoptotic cells was calculated from the sum of apoptotic cell pixels divided by the sum of all cell pixels for each field of view. Error displayed is the standard deviation from between two and four biological replicates.

#### Fluorometric Cholesterol Oxidase Experiments and Synthetic Small Unilamellar Vesicle Preparation

Synthetic small unilamellar vesicles (SUVs) were prepared by dissolving 10 mg l-α-phosphatidylcholine (Sigma; catalog no.: P3556) in 100 μl chloroform in a glass vial, followed by removal of solvent under vacuum at room temperature for 1 h. For experiments using cholesterol-containing SUVs, 0.74 mg of cholesterol (Sigma; catalog no.: C8667) was mixed with 10 mg l-α-phosphatidylcholine prior to removal of chloroform solvent. Following solvent removal, 100 μl PBS was added and a microtip sonicator was inserted to perform sonication at 70% power, 10 pulses, and 5 s on/off at room temperature. SUVs in suspension were brought to a volume of 1 ml with addition of PBS. The resulting SUVs in suspension were used at a dilution of 100-fold in subsequent cholesterol oxidase reactions. Cholesterol oxidase reactions were performed using reagents from the Amplex Red Cholesterol Assay Kit (Thermo Fisher Scientific; catalog no.: A12216), where each reaction was performed in 50 μl volumes using 0.5 μl SUV solution, 0.05 μl cholesterol oxidase solution, 0.05 μl horseradish peroxidase solution, 0.05 μl Amplex Red/dimethyl sulfoxide made according to manufacturer’s instructions, and the indicated CBD concentrations in PBS. Reaction volume was brought to 50 μl using PBS. In cases where SUVs were not used, either 1 μg/reaction 25-OH cholesterol or 5 μg/reaction methyl-beta-cyclodextrin (MBCD):cholesterol (1:2) was substituted for SUVs. MBCD:cholesterol was prepared as previously described (50). Cholesterol oxidase reactions were performed in Corning 384-well optical imaging plates (catalog no.: 3985) in an ImageXpress MicroXL widefield fluorescence microscope using the TRITC filter sets, where 1 ms exposure time images were taken of each well 20 μm above the well bottom every 10 min for 5 h at 37 °C, using a 10× objective. Images were flat-field corrected, and the sum of fluorescence intensity across all 540 × 540 pixels was calculated using custom MATLAB scripts that are available upon request. Product formation of Amplex Red was found to be linear within 0 to 1 h, and data between *t* = 0 and *t* = 1 h were used to calculate the average rate of increase in TRITC fluorescence using Microsoft Excel. Displayed error bars represent the standard deviation of three or more replicate reactions.

#### Fluorescence Recovery After Photobleaching Experiments Using Synthetic Membranes

The formulation and techniques to create SUVs were repeated with addition of 108 μg of 22-NBD-cholesterol to l-α-phosphatidylcholine and cholesterol prior to the solvent removal step described for preparation of SUVs. A 1:5 dilution of NBD-cholesterol containing SUV suspension:PBS was added to each well of glass bottom 96-well plates (MatriPlate; catalog no.: MGB096-1-2-LG-L) such that each well contains 100 μl of diluted SUV suspension. About 96-well plates were exposed to centrifugation for 20 min at 2000 rcf using a swinging bucket rotor at room temperature. A microtip sonicator was inserted into each well to perform sonication at 20% power, 20 pulses, 2 s on/off at room temperature. The contents of each well were washed three times with 150 μl of PBS, and subsequent experiments were performed with 250 μl PBS containing ethanol vehicle, 60 μM CBD, and/or 20 μM docosahexaenoic acid (DHA). CBD and DHA were incubated in wells for 1 h at room temperature prior to imaging and fluorescence recovery after photobleaching (FRAP) experiments using a Nikon A1R microscope. Photobleaching was performed using Nikon Elements software with the following parameters: frame rate 250 ms, 100% power 488 laser for photobleaching for 250 ms, and optical settings for FITC. Analysis was performed using ImageJ (NIH). All trends were normalized by division of mean intensity within the photobleached region to a region of identical size remote from the photobleached region. Error bars indicate the standard error of the mean from three replicates.

#### Seahorse Extracellular Flux Analysis

Oxygen consumption rate and extracellular acidification rate were measured using the SeahorseXF^e^24 Extracellular Flux Analyzer and the Agilent Seahorse XF Cell Energy Phenotype Test Kit. Cells were plated at 2 × 10^4^ cells per well in XF^e^24 microplates. Cells were treated with either 20 μM CBD or ethanol as a vehicle control either 24 h or 2 h prior to assaying. The day of the assay, cells were washed with an assay medium containing 20 μM CBD or vehicle and placed at 37 °C in a CO_2_-free incubator for 1 h. About 1 μM oligomycin and 1 μM carbonyl cyanide *p*-(trifluoromethoxy)phenylhydrazone were injected by the Seahorse analyzer as oxygen consumption rate and extracellular acidification rate were measured per manufacturer’s protocol.

#### Filipin Permeabilization Assay

About 40,000 SK-N-BE(2) cells were seeded into each well of a 96-well plate. After 18 h, cells were exposed to vehicle or CBD for 24 h, followed by 1 h incubation with Filipin (Sigma; catalog no.: F9765) in the presence of Hoescht 33258 and propidium iodide. Cells were imaged using DAPI and TRITC filter sets on an ImageXpress MicroXL microscope. TRITC fluorescence was quantified as the sum of pixel intensity above background after flat-field correction.

## Results

### FRET-based Sensor Array Reveals CBD Response Dynamics

To identify molecular events initiated by CBD treatment, we performed temporal multiomic profiling of CBD-treated human neuroblastoma cells. The dynamics of metabolite, RNA, and protein changes in response to drug perturbation can span time scales ranging from seconds to days, presenting a challenge for selecting appropriate time points in multiomic analysis. To identify the optimal time points and CBD dose for multiomic profiling, we used high-content imaging to monitor a panel of human cell lines (SK-N-BE(2) neuroblastoma and HaCaT keratinocyte cells) expressing FRET sensors. Transgenic lines were generated, each expressing a genetically encoded FRET biosensor gene capable of reporting a cellular activity (51). Sensors were selected to profile a broad range of activities, including abundance changes in metabolites and secondary messengers, as well as kinase and protease activities (supplemental Table S1).

**Fig. 1:**
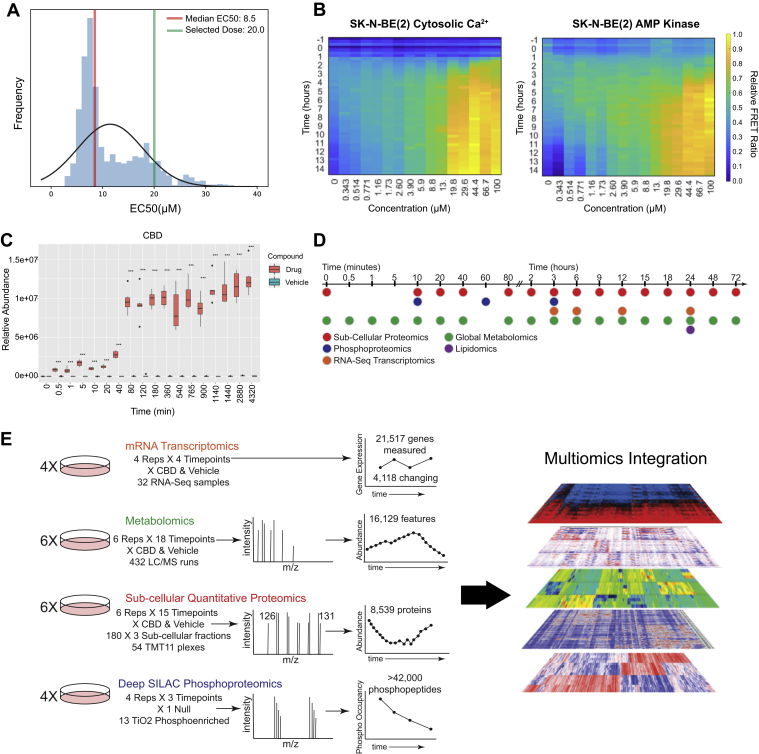
**FRET biosensor screening strategy for dose and time selection of multiomic CBD perturbation analysis.***A*, EC_50_ distribution of CBD treatment across all sensors and time points (see also supplemental Table S1 for list of sensors). Dose–response curves were fit to determine EC_50_ values (*R*^2^ values >0.75). *B*, heatmaps of FRET biosensor responses to CBD treatment for cytosolic Ca^2+^ and AMPK activity in SK-N-BE(2) cells, displaying FRET ratio over time at CBD doses from 343 nM to 100 μM. *C*, relative abundance of CBD over time from metabolomic profiling of SK-N-BE(2) cells treated with 20 μM CBD. *D* and *E*, time-course schematic of multiomic experimental strategy. AMPK, AMP-activated protein kinase; CBD, cannabidiol.

FRET ratios were measured in a time course following cells treated with vehicle or CBD across a range of doses from 0 to 100 μM (supplemental Fig. S1). At each time point, we fit a log-logistic function with FRET ratio data to estimate EC_50_ values for CBD and quantify the dose dependency for each sensor over time. We found that cytosolic calcium abundance, plasma membrane charge, AMPK activity, extracellular signal–regulated kinase activity, and glucose abundance exhibited the most significant dose-dependent changes (*R*^2^ ≥ 0.75). An EC_50_ distribution was generated from CBD dose responses across all time points and biosensors and displayed a median of 8.5 μM for SK-N-BE(2) cells. However, at early time points, a minimum of 20 μM CBD was required to activate FRET sensors for which an EC_50_ could be estimated, including cytosolic calcium, AMPK activity, and plasma membrane charge (Fig. 1*A*). SK-N-BE(2) cells displayed a higher degree of dose dependency in FRET sensor activation over time relative to HaCaT cells (supplemental Fig. S1). We therefore selected SK-N-BE(2) cells treated with 20 μM CBD for subsequent multiomic experiments.

Biosensor screening revealed that CBD led to activation of a diverse spectrum of cellular activities. After treatment with 20 μM CBD, the earliest FRET sensor activities observed were increased cytosolic calcium at 3 h, followed closely by AMPK activity (Fig. 1*B*). AMPK can be activated by distinct mechanisms: through allosteric binding of AMP, as a result of increased cellular abundance of AMP relative to ATP, or through phosphorylation-dependent activation by Ca^2+^/calmodulin-dependent protein kinase kinase β (CaMKKβ) or STK11 (also known as LKB1) (52, 53, 54). CaMKKβ increases the activity of AMPK in a calcium-dependent manner through direct interactions with its kinase domain, driving downstream secondary calcium signaling events (53, 55). Our observation that CBD treatment leads to increased cytosolic calcium is consistent with previous reports of CBD driving an increase in cytosolic calcium through TRPM8 (transient receptor potential cation channel subfamily M [melastatin] member 8), TRPV receptors, or voltage-dependent T-type receptors (17, 23).

We next monitored the CBD cellular uptake kinetics in SK-N-BE(2) cells. The relative abundance of intracellular CBD was quantified by MS in a time course from 30 s to 72 h. CBD was detected in cells as early as 30 s but did not reach steady state until 80 min post-treatment (Fig. 1*C*). Based on this time course, we performed a set of multiomic experiments to examine the temporal response of SK-N-BE(2) cells to CBD treatment, from minutes to days, using global metabolomics, lipidomics, phosphoproteomics, subcellular proteomics, and transcriptomics (Fig. 1*D*). This effort resulted in the detection of >42,000 phosphorylated peptides, 8359 proteins, 21,517 gene transcripts, and 16,129 metabolic features (Fig. 1*E*).

### CBD Activates AMPK Signaling and Downstream Substrate Phosphorylation

**Fig. 2:**
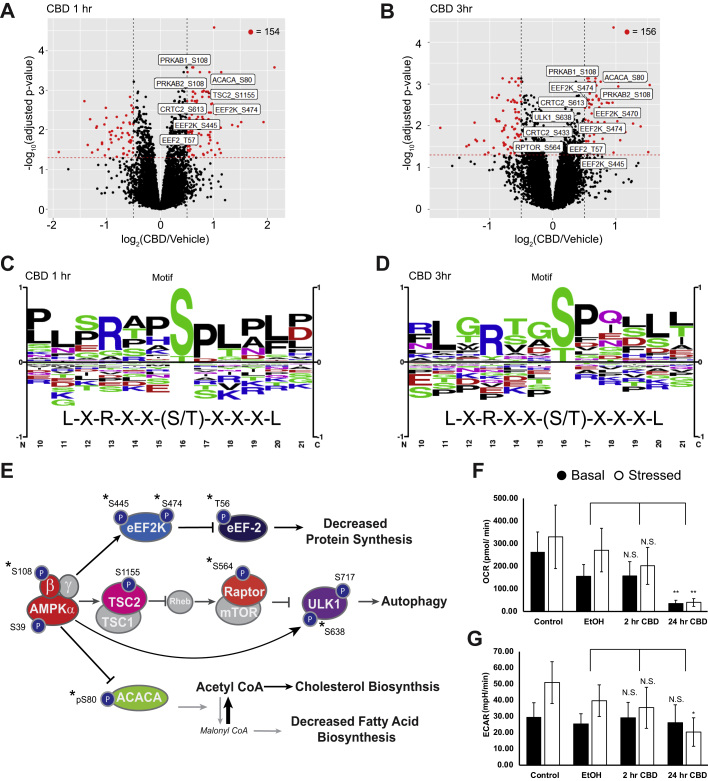
**CBD increases phosphorylation of AMPK signaling proteins at early time points.***A* and *B*, volcano plots of quantified phosphorylation sites at 1 and 3 h post-treatment with 20 μM CBD, showing significance of differential change on *y*-axis and log_2_(CBD/DMSO) ratio on *x*-axis. *Red*: adjusted *p* < 0.05, |log_2_(CBD/DMSO)| >0.5. Sites on AMPK proteins involved in AMPK signaling with adjusted *p* value < 0.05 are annotated in *white boxes*. *C* and *D*, phospho-motif enrichment from phosphorylation sites identified as significantly changing in CBD-treated cells *versus* vehicle control at 1- and 3-h after CBD treatment. The AMPK averaged motif is displayed beneath the sequence logo (57). *E*, overlay of significantly changing phosphorylated sites onto an AMPK signaling diagram. *Asterisks* signify phosphorylation sites with a known upstream kinase. *F* and *G*, seahorse extracellular flux measurement of oxygen consumption rate (OCR) and extracellular acidification rate (ECAR). ∗*p* < 0.05; ∗∗*p* < 0.01. Stressed condition: oligomycin treatment (1 μM)/FCCP treatment (1 μM). AMPK, AMP-activated protein kinase; CBD, cannabidiol; DMSO, dimethyl sulfoxide; FCCP, carbonyl cyanide *p*-(trifluoromethoxy)phenylhydrazone.

We performed quantitative phosphoproteomics to quantify changes in site-specific phosphorylation events in response to CBD treatment at 10 min, 1 h, and 3 h, using stable isotope labeling of amino acids in cell culture (56). At 10 min, only five significantly changing phosphorylation sites were observed (q < 0.05 and |log2 ratio| >0.5) (supplemental Fig. S2*A*). However, the number of significantly changing sites increased to 154 by 1 h (Fig. 2*A*), mirroring the kinetics of CBD uptake into cells between 40 and 80 min (Fig. 1*C*). At both 1 h and 3 h time points, significantly changing phosphorylation sites were enriched in known effectors of AMPK signaling. (Figs. 2*A*, 2*B* and S2*B*). The canonical phosphorylation motif of high-confidence AMPK substrates has been identified as L-X-R-X-X-(pS/pT)-X-X-X-L0 (57, 58, 59). We found that AMPK motifs were significantly enriched in CBD-responsive phosphorylation sites at 1 and 3 h, including L-X-R-X-X-pS and R-X-X-pS-X-X-X-L (Fig. 2, *C* and *D*, and supplemental Fig. S2, *C* and *D*).

Of the CBD-responsive phosphorylation sites on proteins involved in AMPK signaling, several are annotated with biological function. We observed increased phosphorylation of S108 within the beta subunit of AMPK at the 1 and 3 h time points. Phosphorylation of S108 drives a conformational change in the AMPK complex, resulting in stabilization of active kinase by preventing dephosphorylation of the activation site, T172 (60). In agreement with increased AMPK activity after 1 h of CBD treatment, we found increased phosphorylation of S80 on acetyl-CoA carboxylase (ACACA), a known AMPK phosphorylation site (Fig. 2*E*) (61, 62)*.* ACACA catalyzes the rate-limiting step of FA synthesis and is deactivated by AMPK phosphorylation of S80. Phosphorylation of ACACA S80 results in reduced conversion of acetyl-CoA into malonyl-CoA, reducing carbon flux through FA synthesis, and increasing catabolic FA β-oxidation (63, 64)*.* In line with these findings, we observed decreased flux of carbon into *de novo* synthesized FAs (supplemental Fig. S2*E*). We found significantly decreased levels of short-chain and medium-chain but not long-chain acylcarnitines in the CBD-treated cells, indicating that FA mobilization is comparable in the two groups but more rapidly fluxed through FA β-oxidation upon treatment with CBD (supplemental Fig S2*F*).

We also identified increased phosphorylation of the translation elongation factor, eukaryotic elongation factor 2 (EEF2), on T56 with CBD treatment at 1 and 3 h. EEF2 T56 phosphorylation is sufficient to inhibit the GTP-dependent ribosomal translocation step during translational elongation, consistent with upstream activation of AMPK and EEF2K (65). Together, these observations predict alterations of both protein and FA synthesis downstream of AMPK activation by CBD.

Agreement between phosphoproteomics and the AMPKAR FRET biosensor data indicates that AMPK is activated by CBD treatment, raising the question of whether AMPK is activated through increased AMP:ATP ratio or by upstream kinases. To test whether CBD treatment acutely alters cellular energy status, we measured the oxygen consumption rate and extracellular acidification rate of CBD-treated cells using a Seahorse extracellular flux assay. Treatment of SK-N-BE(2) cells with 20 μM CBD led to decreased levels of basal oxygen consumption by 24 h, with little change at 2 h postdrug treatment (Fig. 2*F*). Basal extracellular acidification rate remained unchanged (Fig. 2*G*). Consistent with these findings, we observed comparable rates of lactate production but decreased carbon flux into tricarboxylic acid cycle metabolites in cells treated with CBD (supplemental Fig. S2*G*). These results suggest that CBD-treated cells have decreased ATP production by mitochondrial respiration with little to no compensation by glycolysis, which may sustain AMPK activation at late time points. While we do not have direct evidence of the mechanism by which AMPK is activated between 1 and 3 h, we hypothesize that in the absence of compromised ATP production at early time points, calcium influx into the cytoplasm may be responsible for activation of AMPK through upstream kinases such as CAMKKβ.

### CBD Upregulates Transcripts and Proteins Involved in Cholesterol Biosynthesis

**Fig. 3:**
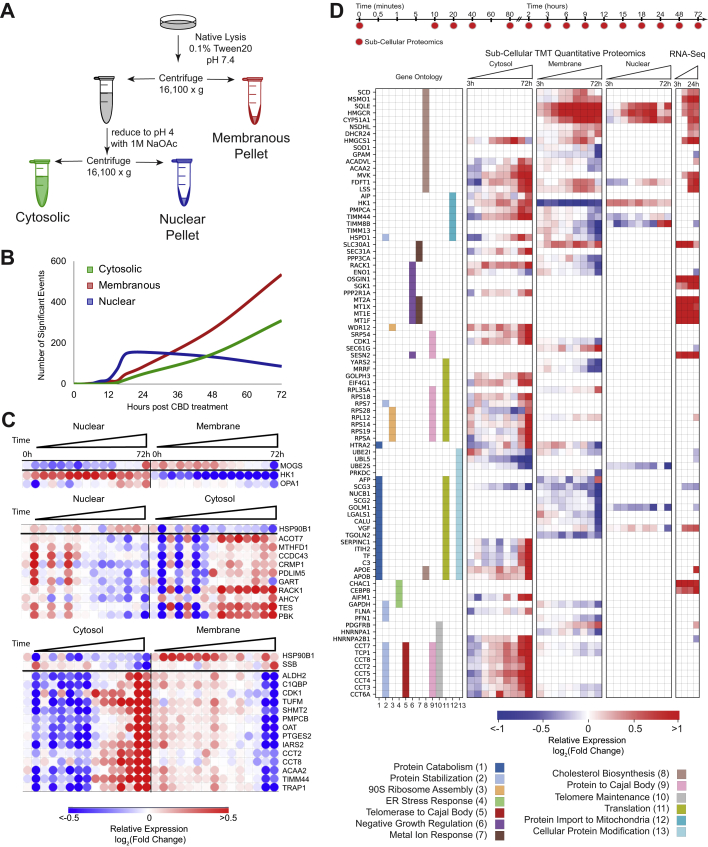
**CBD treatment upregulates cholesterol biosynthesis enzymes and translocation of metabolic proteins.***A*, compositionally distinct subcellular proteomic fractions were fractionated by differential centrifugation and pH. The “cytosolic” fraction is enriched in soluble protein, the “nuclear” fraction is enriched in insoluble subnuclear compartments: condensed chromosome, spindles, spliceosomal complex, and so on, and the “membrane” fraction is enriched in membrane- and mitochondrial-related proteins. (supplemental Fig. S3, *C* and *E*). *B*, frequency of significantly changing proteomic events over time. *C*, anticorrelated proteins between proteomic fractions over time. PCA dimensionality reduction was used to decrease the impact of noisy signal contribution. Correlation between fractions, *r* < −0.8 was required. A large proportion of proteins listed previously are known to compartmentalize in the mitochondria indicating protein shuttling or mitochondrial detachment/attachment (supplemental Fig. S3*F*). *D*, proteins and mRNA transcripts that change significantly with CBD and map to the indicated Gene Ontology annotations that showed significant enrichment of differential proteins (see the Experimental procedures section). CBD, cannabidiol; PCA, principal component analysis.

To identify time-dependent proteome changes in subcellular compartments, we developed a pH-dependent cell fractionation scheme using differential centrifugation (Fig. 3*A*). The resulting “cytosolic” fraction is enriched in soluble proteins from the cytosol, nucleus, and various luminal compartments (*e.g.*, mitochondria) (supplemental Fig. S3*A*). The first insoluble fraction, labeled as “membrane,” is enriched in proteins from mitochondrial and plasma membranes, whereas the second insoluble fraction is highly enriched in insoluble nuclear components, including condensed chromatin, spindles, and nuclear speckles (supplemental Fig. S3, *B* and *C*). Principal component analysis of these fractions revealed three compositionally distinct portions of the proteome, with each of these fractions exhibiting a time-dependent separation in response to CBD treatment. (supplemental Fig. S3, *D* and *E*). However, the membranous and nuclear fractions remain very similar in principal component analysis space until 12 h and later time points, suggesting relatively slow kinetics of protein regulation in response to CBD. Consistent with this observation, the frequency of significant events across fractions is limited at time points prior to 12 h but increases dramatically to hundreds of proteins at time points between 15 and 72 h (Fig. 3*B*).

A protein that translocated between cellular compartments in response to CBD would be expected to have anticorrelated time courses in those subcellular fractions. To identify potential translocation events, we calculated the Pearson’s correlation coefficient between the temporal profiles of each of that protein’s subcellular fractions. We found 30 proteins with highly anticorrelated subcellular profiles (Fig. 3*C*). Notably, hexokinase 1 (HK1) decreased in the membrane fraction and increased in the nuclear fraction (Figs. 3*C* and S3*F*). HK1 detachment from the outer mitochondrial membrane attenuates conversion of the HK1 substrate glucose to glucose 6-P, decoupling glycolysis from mitochondrial respiration, and can alter the overall balance of energy metabolism in the cell (66, 67, 68). Consistently, CBD-treated cells exhibit reduced levels of glucose-6-phosphate at later time points (supplemental Fig. S3*G*). This potential translocation event is consistent with decreased cellular respiration in response to CBD treatment (Fig. 2*F*) and previous reports of CBD-induced mitochondrial dysfunction in neuroblastoma cells (69).

To identify CBD-dependent changes in mRNA transcript abundance, we performed RNA-Seq, comparing SK-N-BE(2) cells treated with 20 μM CBD or vehicle for 3, 6, 12, and 24 h. We identified 4118 differentially expressed transcripts in CBD-treated cells that were significant in at least one time point (*q* < 0.01). About 204 of these genes displayed transcript abundances with a |log2 ratio| ≥1 (Fig. 1*E*). To identify potential transcription factor specific responses that explain mRNA transcript changes, we performed upstream regulator analysis on significantly changing transcripts (70). The most enriched transcription factors for increasing transcripts shared oxidative stress as a stimulus and included *ATF4* (activated transcription factor 4), *NFE2L2*, and *SP1* (supplemental Fig. S3*H*) (71, 72, 73, 74). CBD-treated cells also showed an accumulation of the principal cellular antioxidant glutathione, consistent with an oxidative stress response in CBD-treated cells (supplemental Fig. S3*G*).

We merged differentially expressed transcript and protein identifications and performed GO enrichment analysis using REVIGO pathway analysis (Fig. 3*D*) (75). CBD-responsive events were enriched in translation, ER stress response, metal ion response, and cholesterol biosynthesis (adjacent *p* < 0.01). While many of these annotations are supported by either the transcriptome or the proteome, dysregulation of cholesterol metabolism is supported by both. Within the cholesterol biosynthesis ontology, 17 proteins displayed significant abundance changes that increased over time, including several key regulatory proteins. The rate-limiting enzyme in cholesterol synthesis, 3-hydroxy-3-methyl-glutaryl-coenzyme A reductase (HMGCR), increased by ∼300% on the protein level across both membranous fractions, with increased transcript abundance at 6 h. Protein levels for superoxide dismutase 1 (SOD1), a negative regulator of HMGCR, decreased by 40%, consistent with derepression of *HMGCR* transcription (76). The enzyme catalyzing the conversion of desmosterol into cholesterol in the terminal step in cholesterol biosynthesis, DHCR24 (24-dehydrocholesterol reductase 24), increased by 43% on the protein level in the “membrane” fraction (Fig. 3*D*). Together, the proteomic and transcriptomic data point to a concerted response in cholesterol homeostasis pathways and suggest that cells upregulate cholesterol biosynthesis capacity when challenged with CBD.

### CBD Treatment Results in Accumulation of Cholesterol Biosynthesis Intermediates and Esterified Cholesterol

**Fig. 4:**
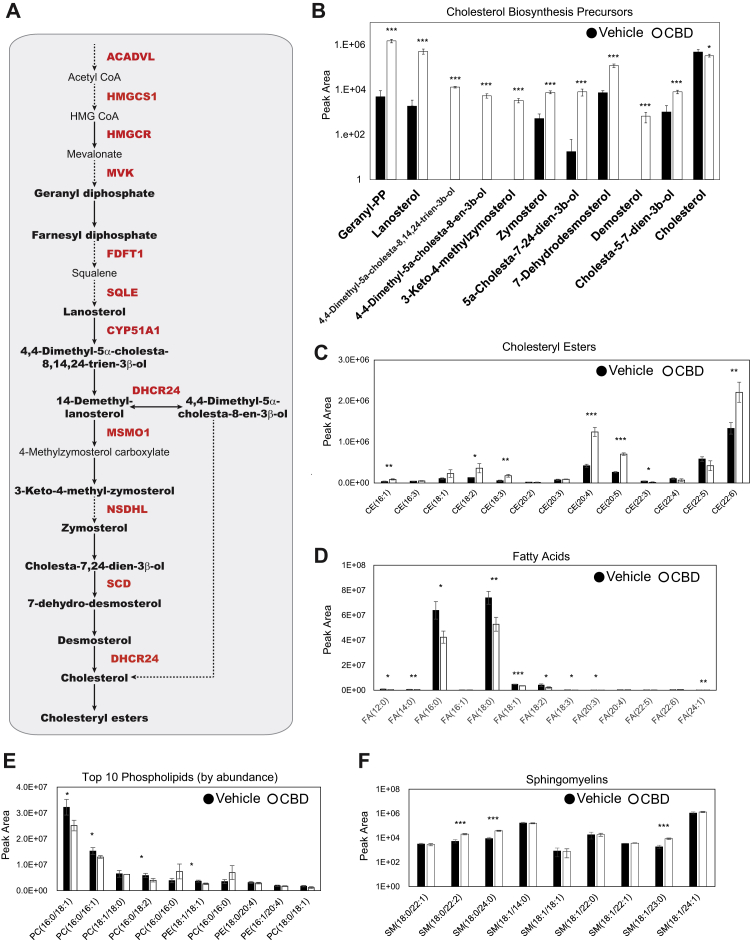
**Cholesterol biosynthesis precursors and cholesterol esters accumulate upon CBD treatment.***A*, pathway diagram of cholesterol biosynthesis. Quantified metabolic intermediates are outlined in *black bold*, and enzymes showing significant increase over time with CBD in proteomic analysis are shown in *bold red* (Fig. 3*D*). *Dashed arrows* indicate multiple intermediate steps that were not identified in the pathway. *B*, d-glucose (U-(13)C_6_) metabolically labeled cholesterol biosynthesis precursors at 24 h post 20 μM CBD treatment. (Student’s *t* test: ∗*p* < 0.05, ∗∗*p* < 0.01, and ∗∗∗*p* < 0.001) (supplemental Fig. S4). *C*–*F*, total abundance of lipids quantified by LC–MS/MS from cell extracts of SK-N-BE(2) cells treated with vehicle or 20 μM CBD. Cholesteryl esters (CEs), fatty acids (FAs), phospholipids, and sphingomyelins (SMs) identified by mass spectrometry are displayed by peak area (supplemental Fig. S4, *D* and *E*). (Student’s *t* test: ∗*p* < 0.05, ∗∗*p* < 0.01, and ∗∗∗*p* < 0.001). CBD, cannabidiol; PC, phosphatidylcholine; PE, phosphatidylethanolamine.

Proteomic and transcriptomic analyses revealed a CBD-induced upregulation of cholesterol biosynthesis machinery. These findings raised the question of whether CBD treatment leads to alterations in lipid and cholesterol metabolism (the latter pathway depicted in Fig. 4*A*). We used MS-based lipidomics to quantify the effect of CBD on lipids and sterols. Vehicle and CBD-exposed cells were labeled with [U-^13^C_6_]-d-glucose for 24 h and harvested using methanol extraction. Cholesterol biosynthetic flux was quantified by MS analysis of ^13^C incorporation into biosynthetic intermediates. We found that cholesterol precursors accumulated in CBD-exposed cells (Figs. 4*B* and S4*A*), whereas labeled and total cholesterol itself decreased modestly (Fig. 4*B* and S4B). This effect of CBD on total cellular cholesterol was confirmed using an Amplex Red cholesterol assay (supplemental Fig. S4*C*).

Internalized cholesterol is stored in lipid droplets after esterification with long-chain FAs by acyl-coenzyme A cholesterol O-acyltransferase enzymes, using long-chain fatty acyl-coenzyme A as the FA donor (77). We detected accumulation of multiple species of cholesteryl esters with various chain lengths and acyl-chain saturation (Fig. 4*C*). Upregulation of cholesterol biosynthesis enzymes, together with increased abundance of metabolic precursors, suggests that CBD leads to increased production and storage of cholesterol esters. Because of the requirement of acyl-coenzyme A precursors in cholesterol esterification (78), we surveyed our dataset for evidence of FA utilization. CBD treatment led to significantly reduced levels of the most abundant FAs including FA (16:0) and FA (18:0), which accounted for a large percentage of the total FA content that was detected (Fig. 4*D*).

To determine if these CBD-dependent changes on cholesterol metabolism resulted in significant changes in membrane composition, we profiled the cellular abundance of all detectable species of phosphatidylcholine and phosphatidylethanolamine from cell extracts. Several of the most abundant phospholipid species displayed a significant reduction in abundance following CBD treatment (Fig. 4*E*). As cholesterol is a critical structural component of cellular membranes, a decrease in total cholesterol levels could alter physical properties of the phospholipid bilayer and require compensation from membrane-ordering lipids. To this end, we surveyed spingomyelins (SMs) in our lipidomics data and found while most species were unchanged, three specific SMs, SM (18:0/22:2), SM (18:0/24:0), and SM (18:1/23:0), were increased upon CBD treatment (Fig. 4*F*). These observed increases suggest a compensatory reorganization within cell membranes in response to the changes in cholesterol biosynthesis and storage. Head groups identified by lipidomics showed further reorganization, with *sn*-glycero-3-phosphaoethanolamine decreasing dramatically and a twofold increase in phosphoethanolamine, a product of sphingosine catabolism *via* the enzyme S1P lyase (supplemental Fig. S4*D*). Together, this evidence suggests that CBD leads to remodeling of the lipidome and perturbation to cholesterol homeostasis pathways (supplemental Fig. S4*E*). To functionally validate that CBD elicits cholesterol storage, we used live-cell confocal microscopy of SK-N-BE(2) cells stained with both fluorescent cholesterol (22-NBD-cholesterol) and the lipid droplet dye, Nile red (supplemental Fig. S4*F*). CBD treatment resulted in slight increase in the average abundance of lipid droplets per cell but did not affect the size of these droplets.

### CBD Increases Storage and Transport of Cholesterol

**Fig. 5:**
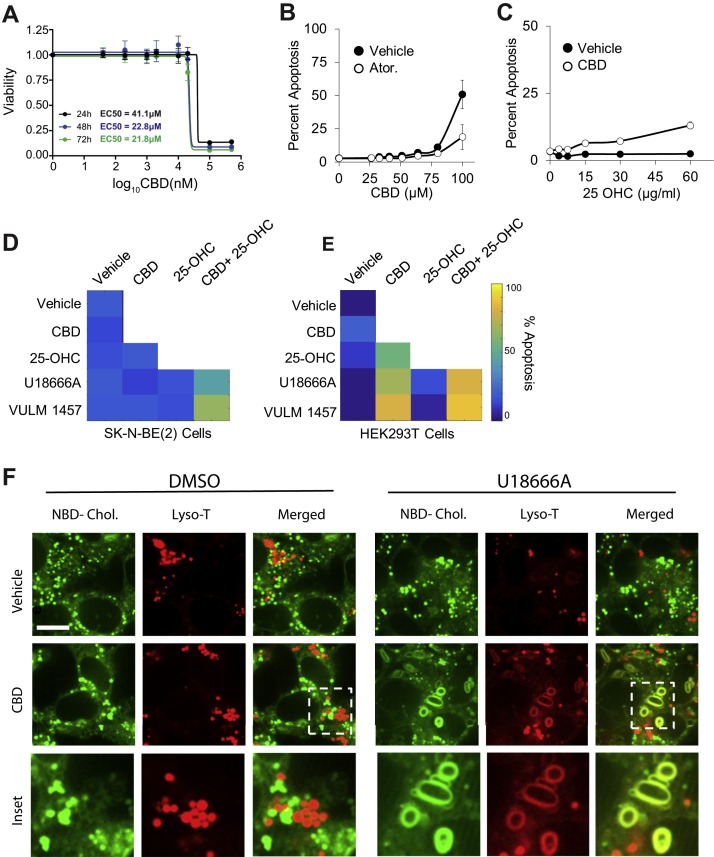
**CBD-induced apoptosis is rescued by inhibitors of cholesterol synthesis and increased by inhibitors of cholesterol transport and storage.***A*, CBD was assessed for cytotoxicity in SK-N-BE(2) cells across increasing doses of CBD at 24, 48, and 72 h by CellTiter-Glo luminescent assay. *B*–*E*, SK-N-BE(2) or HEK293T cells were assessed for apoptosis at 24 h using live-cell microscopy using a resazurin-based fluorometric cell viability stain. Cells were treated with 10 μM atorvastatin and exposed to increasing doses of CBD in *B*. About 20 μM CBD and exposure to increasing doses of 25-OH cholesterol in *C*, and combinations of 20 μM CBD, 10 μM U18666A, 5 μM VULM, and 15 μg/ml 25-OH cholesterol at 48 h in *D* and *E*. Apoptosis displayed in a heatmap for each condition (supplemental Fig. S5). *F*, live-cell confocal microscopy of SK-N-BE(2) with NBD-cholesterol (*green*) and a lysosomal dye Lyso-T (*red*). Cholesterol subcellular distribution was examined upon exposure of cells to 20 μM CBD and/or 10 μM U18666A. The scale bar represents 3 μm. CBD, cannabidiol; HEK293T, human embryonic kidney 293T cell line; 25-OH, 25-hydroxy.

Alterations in cholesterol abundance can lead to severe cellular phenotypes that include mitochondrial dysfunction and apoptosis (79), whereas disruption in cholesterol trafficking is a hallmark of Niemann–Pick disease type C (80). To explore the phenotypic implications of the disruption of cholesterol homeostasis of CBD, we first tested if CBD induced cell death. Dose–response analysis of cell viability revealed that 50% of SK-N-BE(2) cells die after 24 h of treatment with 40 μM CBD (Fig. 5*A*). SK-N-BE(2) cells were then exposed to increasing concentrations of CBD in the presence or in the absence of the cholesterol biosynthesis inhibitor, atorvastatin, and analyzed for apoptosis using CellEvent caspase 3/7 dyes and live-cell fluorescence imaging. At 15 h, 100 μM CBD leads to apoptosis of 50% of SK-N-BE(2) cells. Cotreatment of SK-N-BE(2) cells with CBD and atorvastatin reduced apoptosis by approximately twofold (Fig. 5*B*). This atorvastatin-dependent rescue of CBD-induced apoptosis was far more pronounced in human HaCaT keratinocytes (Fig. S5*A*), which are known to be highly sensitive to cholesterol perturbation (81). Furthermore, CBD-treated SK-N-BE(2) and HaCaT cells show an increase in apoptosis with increasing concentrations of a soluble form of cholesterol, 25-hydroxycholesterol (25-OHC) (Figs. 5*C*, and S5*B*). Together, these results show that CBD sensitizes cells to apoptosis when challenged with excess cholesterol, either from endogenously synthesized or exogenous pools.

We next determined the dependency of CBD-induced apoptosis on cholesterol transport and storage. We measured apoptosis in SK-N-BE(2) and human embryonic kidney 293T (HEK293T) cells treated with CBD and sublethal doses of 25-OHC (15 μg/ml), in combination with a cholesterol transport inhibitor (Niemann–Pick C1 [NPC1] inhibitor U18666A, 10 μM) or an inhibitor of acyl-coenzyme A cholesterol O-acyltransferase, an enzyme required for esterification and intracellular storage of cholesterol (VULM 1457, 5 μM). Both compounds sensitized cells to apoptosis when CBD was present, which was more pronounced when cells were also challenged with 25-OHC (Fig. 5, *D* and *E*). VULM and U18666A treatment alone did not lead to increased apoptosis. These results demonstrate that interfering with cholesterol transport or cholesterol storage sensitizes cells to CBD-induced apoptosis.

One possible explanation for why CBD sensitizes cells to inhibitors of cholesterol trafficking and storage is that CBD increases the rate of cholesterol transport from the plasma membrane through the endosomal–lysosomal pathway. In support of this hypothesis, we observed increased abundance of apolipoproteins B and E in the cytosol-enriched fraction of the proteome. Apolipoproteins B and E are lipoprotein components of cholesterol-containing low-density lipoprotein particles required for cellular uptake of cholesterol (Fig. 3*D*). When cholesterol import through low-density lipoprotein receptors is activated, and 25-OHC is supplied in excess, the inability to efficiently store cholesterol may cause accumulation of cholesterol in organelles that normally maintain low levels. To examine this possibility, we visualized lysosomes (LysoTracker dye) and cholesterol (NBD-cholesterol) in vehicle and CBD-treated SK-N-BE(2) cells with live-cell confocal microscopy.

In cells treated with either CBD or cholesterol transport inhibitor, puncta stained with LysoTracker or NBD-cholesterol showed distinct spatial separation within cells. In contrast, cotreatment with CBD and U18666A led to formation of enlarged membranous features costained with LysoTracker and NBD-cholesterol (Fig. 5*F*). Morphologically similar structures have been reported in models of Niemann–Pick disease type C. In this case, cholesterol accumulates in enlarged lamellar inclusions with components of lysosomes and endosomes, leading to a toxic cycle of enhanced cholesterol synthesis and intracellular accumulation (82, 83). These data support a model where CBD increases transport of cholesterol from plasma membrane through the endosomal–lysosomal pathway to intracellular compartments where it is esterified and sequestered, while escaping ER-resident cholesterol sensing machinery (84, 85).

### CBD Incorporates Into Membranes, Alters Cholesterol Accessibility, and Impairs Lateral Diffusion

**Fig. 6:**
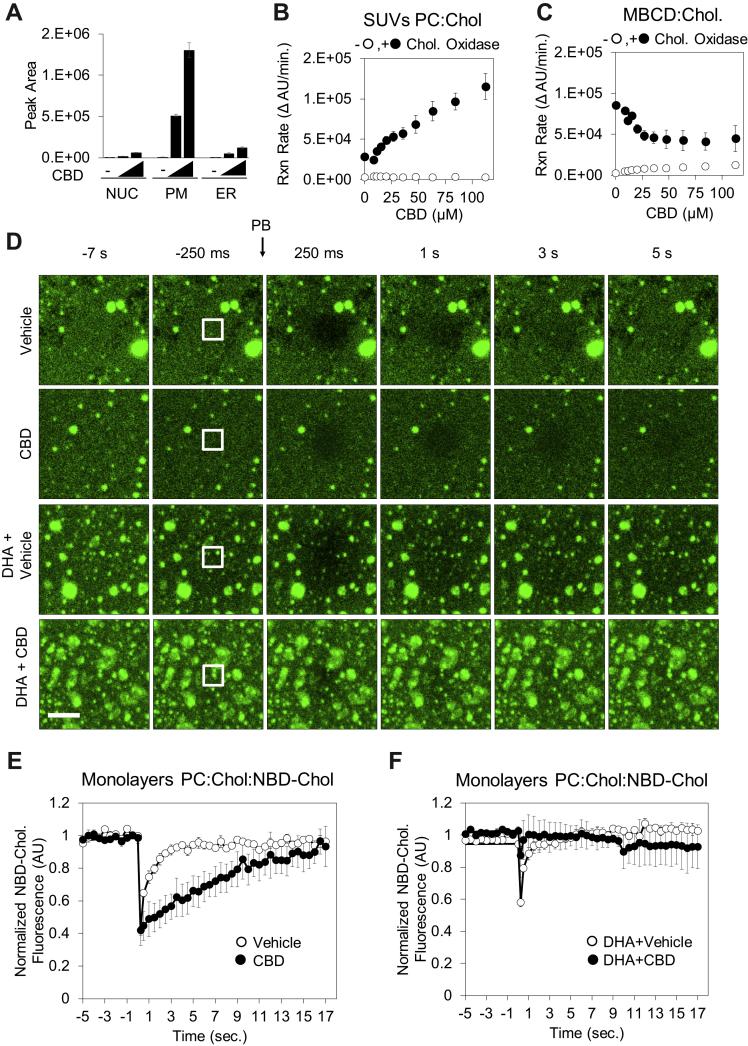
**CBD incorporates into membranes, increases cholesterol accessibility, and reduces lateral diffusion of cholesterol.***A*, ethanol extracts of subcellular fractions of SK-N-BE(2) cells exposed to 0, 20, and 40 μM CBD for 24 h were analyzed for CBD using LC–MS. *B*, synthetic small unilamellar vesicles (SUVs—molar ratio is phosphatidylcholine:cholesterol:NBD-cholesterol: 78:20:2 [n/n%]) were used as a source of cholesterol in a fluorogenic cholesterol oxidase reaction to determine the effect of CBD on initial reaction rate. *C*, identical experiments were performed with cholesterol complexed to methyl beta cyclodextrin (MBCD), without SUVs present (*D*). Synthetic membrane monolayers containing NBD-cholesterol were adsorbed to borosilicate glass and used in fluorescent recovery after photobleaching (FRAP) experiments following exposure to either CBD (60 μM) and/or DHA (20 μM). The scale bar represents 2.5 μm. Quantified fluorescence recovery after photobleaching is displayed in (*E*) and (*F*) (n = 3). CBD, cannabidiol; DHA, docosahexaenoic acid.

Subcellular fractionation of SK-N-BE(2) cells treated with CBD for 24 h showed that CBD is concentrated primarily at the plasma membrane, with lower levels detected in ER and nuclear membranes (Fig. 6*A*). Because of this localization, and the CBD-induced changes in cholesterol homeostasis, we hypothesized that CBD may alter cholesterol availability to sensing and trafficking proteins within the plasma membrane. To measure the effect of CBD on cholesterol accessibility, we measured the enzymatic oxidation rate of cholesterol to 5-cholesten-3-one by cholesterol oxidase in small unilamellar vesicles (SUVs). Cholesterol oxidase has been shown to sense alterations of lipid bilayer structure and cholesterol accessibility (86) and could reveal CBD-dependent alterations in cholesterol orientation in membranes. Titration of CBD into cholesterol-containing SUVs increased the initial reaction rate of cholesterol oxidase in a manner proportional to CBD concentration (Figs. 6*B* and S6*A*).

The dose-dependent increase in cholesterol oxidase activity by CBD requires cholesterol localized in membranes. Freely soluble 25-OHC and soluble complexes of cholesterol and MBCD showed no dose dependence on CBD (Figs. 6*C* and S6*B*). We repeated this experiment in a complex membrane environment using vesicles derived from ER membranes and again observed a concentration-dependent increase in cholesterol oxidase activity in response to CBD (supplemental Fig. S6*C*). Together, these data provide evidence that CBD incorporates into membranes and alters cholesterol accessibility, likely by altering cholesterol orientation within the membrane to make the hydroxyl moiety more solvent accessible.

The ability of cholesterol oxidase assays to reveal alterations in lipid order has been previously demonstrated in studies noting that cholesterol oxidase can preferentially target caveolar domains, a specialized type of lipid-ordered domain (87, 88). A hallmark of increased lipid order is a decrease in lateral diffusion of lipids (89, 90). To determine if CBD contributes to changes in lipid order, we measured the effect of CBD on the lateral diffusion of fluorescently labeled cholesterol (NBD-cholesterol) in synthetic membrane monolayers. SUVs containing 20% (n/n%) of cholesterol and 2% (n/n%) NBD-cholesterol were deposited on glass-bottom multiwell imaging plates, followed by ultrasonification. Recovery kinetics of fluorescent cholesterol were monitored in the presence of vehicle or CBD using FRAP (Fig. 6*D*). CBD significantly reduced the recovery of fluorescence in the photobleached monolayer area relative to vehicle control (Fig. 6, *D* and *E*), suggesting that CBD slows the lateral diffusion of fluorescent cholesterol. This effect of CBD on lateral diffusion could be rescued with simultaneous treatment of the DHA, a known disrupter of lipid order (Fig. 6, *D* and *F*).

Our FRAP experiments demonstrate that DHA and CBD have opposing effects on the lateral diffusion of fluorescently labeled cholesterol in synthetic membranes. However, it remains unclear how the biophysical effects of CBD and DHA on cholesterol impact cellular physiology. Esterification of DHA into membrane phospholipids results in remodeling of sphingolipid/cholesterol-enriched lipid rafts, a known hub for apoptosis signaling (91, 92). To determine whether CBD and DHA also have opposing effects in a cellular context, we quantified the effect of CBD and DHA on apoptosis. DHA treatment induced apoptosis in both HEK293T and SK-N-BE(2) cells in a dose-dependent manner (supplemental Fig. S6, *D* and *E*), consistent with previous studies (93, 94, 95, 96). Importantly, this DHA-induced apoptosis proved to be cholesterol dependent, as simultaneous treatment with DHA and the cholesterol-sequestering agent, MBCD, delayed apoptosis in HEK293T cells and fully rescued apoptosis in SK-N-BE(2) cells (supplemental Fig. S6, *D* and *E*). Similarly, CBD treatment (6.25 μM) rescued the apoptotic effects of DHA in both HEK293T and SK-N-BE(2) cells at 48 h (supplemental Fig. S6, *D* and *E*). These data indicate that CBD and DHA have opposing effects on cellular membrane structure and induction of apoptosis, both of which are cholesterol dependent, but the connection between these two processes remains unclear.

Consistent with increased cholesterol accessibility, we found that CBD sensitized cells to permeabilization by the chemical agent filipin. Filipin is a highly fluorescent probe known to bind cholesterol and disrupt nearby lipid ordering (97), resulting in permeabilization of membranes. Cells pretreated with 20 μM CBD for 24 h were preferentially permeabilized by filipin, relative to vehicle control (supplemental Fig. S6*F*). These data suggest that CBD either directly increases cholesterol availability to filipin or destabilizes the membrane, thereby contributing to the membrane disruption effects of filipin.

### CBD Elicits Distinct Phenotypic Profile From Structural Analogs

**Fig. 7:**
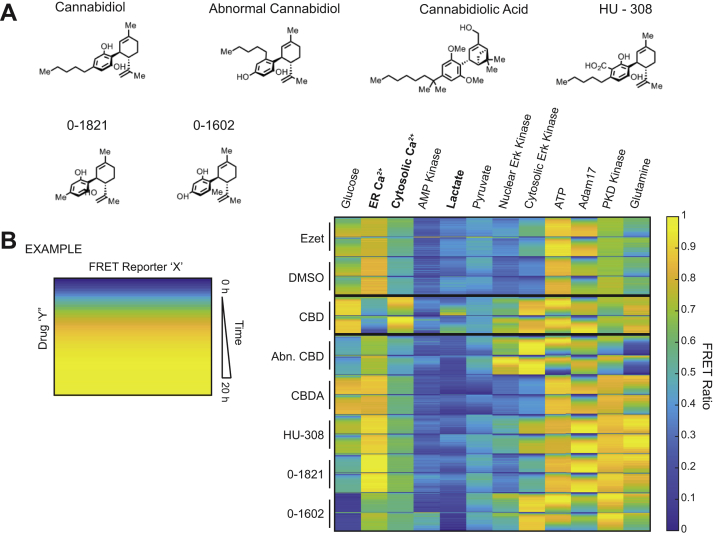
**CBD analogs highlight CBD-specific cellular phenotypes.***A*, chemical structures of CBD and the CBD analogs: abnormal CBD, cannabidiolic acid, HU-308, 0-1602, and 0-1821. *B*, genetically encoded FRET biosensors for diverse biochemical pathways were delivered to SK-N-BE(2) cells. These SK-N-BE(2) cell lines were treated with 20 μM of each small molecule from (*A*) and 10 μM ezetimibe, respectively. FRET ratios were measured in 20 min increments from 0 h through 20 h. Two replicates of each drug or vehicle treatment are displayed. CBD, cannabidiol.

We next sought to evaluate whether dysregulation of cholesterol homeostasis could phenocopy the CBD-driven events that were observed in our FRET biosensor array in Figures 1*B* and S1*A*. To this end, we profiled the NPC1-like I protein inhibitor, ezetimibe, in SK-N-BE(2) cells against CBD and a panel of structurally similar molecules including cannabidiolic acid, abnormal-CBD, as well CBD analogs HU-308, 0-1602, and 0-1821 (Fig. 7*A*). Ezetimibe attenuates the interaction of NPC1L1 with the adaptor protein 2–clatherin complex that is required for cholesterol import, effectively disturbing the cholesterol levels of the cell (98). However, this molecule failed to elicit significant changes in the FRET sensor ratios that were measured in SK-N-BE(2) cells and displayed a profile similar to that of a vehicle control (Fig. 7*B*). Lack of correlation between ezetimibe and CBD suggests that early events observed in proteins, RNA, and phosphorylation are likely not dependent on cholesterol dysregulation and highlights the multipronged mode of action that CBD has on intracellular signaling.

From this effort, we found CBD-specific events and changes in FRET sensor ratios that were shared between multiple analogs alike. Of note, both the chronic rise in cytosolic calcium and depletion of ER-localized calcium were specific to CBD, implicating the previously observed calcium abundance increase with release by the ER (Fig. 7*B*). Calcium concentration is tightly regulated in the ER, and changes in these levels can elicit an ER stress response, the unfolded protein response, and changes in mitochondrial metabolism, which are consistent with protein ontologies observed in Figure 3*D*, (99). FRET reporters for extracellular signal–regulated and protein kinase D activity were observed in both CBD and abnormal CBD, whereas the robust change in glucose abundance in CBD-treated cells was only seen for cannabidiolic acid. It will be important to evaluate how CBD-specific calcium modulation could contribute to clinical outcomes in future studies.

## Discussion

Although clinical and preclinical evidence point to CBD as a promising therapeutic compound for epilepsy, the cellular targets that mediate its effects in humans remain unclear. In this study, we found that CBD elicited pleiotropic effects on the proteome, transcriptome, and metabolome of human cells. Our data suggest that CBD integrates into cellular membranes and alters cholesterol orientation within the phospholipid environment. Partitioning of CBD into model membranes decreased lateral diffusion of cholesterol, altered cholesterol accessibility, predicting that CBD may alter the biophysical properties of cellular membranes, with consequent effects on diverse membrane proteins and their downstream targets.

We found that CBD treatment led to increased cytosolic calcium within 2 h in human neuroblastoma and keratinocyte cells. AMPK activity followed the observed increase in calcium, suggesting upstream activation of AMPK by the calcium-dependent kinase CAMKKβ. Compromised ATP generation by mitochondrial respiration may sustain AMPK activation after 24 h, as suggested by our Seahorse analysis. Increased AMPK activity, and increased phosphorylation of its substrate ACACA, predicted reduced FA synthesis and altered acetyl-CoA metabolism, which was confirmed by metabolomics analysis. Upregulation of cholesterol biosynthesis on proteome, transcriptome, and metabolomic levels occurred as early as 3 h and was sustained up to 72 h. As acetyl-CoA is an early precursor of cholesterol, the increase in cholesterol biosynthesis precursors is consistent with acetyl-CoA supporting this flux. Parallel to upregulation of cholesterol biosynthesis, we hypothesize that increased cholesterol import may occur through the low-density lipoprotein receptor–endocytic pathway, resulting in increased transport of cholesterol through endosomal–lysosomal trafficking. Increased stress on cholesterol trafficking and regulatory processes, combined with compromised cellular energetics driven by CBD, may contribute to increased apoptosis in CBD-treated cells.

### Concordance of Multiomic Data Points to CBD Disruption of Cholesterol Homeostasis

Integration of our transcriptomics, metabolomics, and proteomics data provided multiple lines of evidence for the disruption of cellular cholesterol homeostasis by CBD. Multiple aspects of cholesterol regulation were dysregulated by CBD:cholesterol biosynthesis (Figs. 3*D* and 4*B*), transport (Figs. 3*D* and 5*F*), and storage (Fig. 4*C*). All three omics analyses provided evidence for perturbed cholesterol biosynthesis. For instance, transcriptomics and proteomics reported transcriptional activation and protein accumulation of the rate-limiting enzyme in the biosynthetic pathway of cholesterol, HMGCR (Fig. 3*D*). Increased HMGCR protein production is a canonical response to decreased cholesterol levels in the ER, where cholesterol is sensed through the sterol regulatory–element binding protein–SREBP cleavage–activating protein axis (100). Consistent with this observation, we found that cholesterol precursors accumulated in CBD-treated cells (Figs. 3*B* and S3*A*), with a modest reduction in total cholesterol (Figs. 3*B*, Fig. S3, *A* and *B*) and a large increase in cholesterol esters.

The upregulation of cholesterol biosynthesis by CBD is paradoxical; cells cultured in cholesterol-replete media with abundant intracellular stores of esterified cholesterol typically downregulate cholesterol biosynthesis by the ER-resident SREBP sensing machinery to maintain homeostatic levels of cellular cholesterol (100). These results suggest that in the presence of CBD, the ER is unable to accurately sense the abundance of cholesterol at the plasma membrane, and as a result, generates an excess of cholesterol that is esterified. One possibility is that CBD prevents cholesterol sensing by GRAM (glucosyltransferase, Rab-like GTPase activator, and myotubularin) domain proteins, which localize to plasma membrane–ER contact sites, bind, and transport specific lipids between the two membranes (101). Within this family, GRAMD1s sense and bind the “accessible” pool of cholesterol that is not currently complexed with other lipid species and transports it to the ER (102). The pools of cholesterol that are either “accessible” or “inaccessible/sequestered” are regulated by the domains they associate with and are frequently driven by SM and phospholipid association (103, 104, 105). CBD-driven alterations of cholesterol orientation and decreased lateral diffusion presented here, together with previously reported CBD-dependent increases in lipid raft stability and size, suggest that the pool of “sequestered cholesterol” is increased in CBD-treated conditions. However, specific investigation of GRAMD1 sensing and partitioning of CBD and cholesterol within the ER membrane will need to be investigated in future studies.

### The Effects On Cholesterol Homeostasis in Therapeutic Applications of CBD

Biosensor and multiomic profiling revealed the activation of a diverse spectrum of cellular activities by CBD; in particular, the disruption of cholesterol and lipid homeostasis that are important for proper membrane function. These results may have broad implications on the mechanistic underpinnings of the clinical effects of CBD. Transmembrane proteins known to be regulated through lipid-ordered domains have been implicated in many of the diseases for which CBD has been proposed as a therapeutic. These include inflammatory disorders, Alzheimer’s disease, and several types of cancer (106, 107, 108, 109, 110). Many targets of CBD proposed to underlie its efficacy as an anticonvulsant are also membrane proteins, including TRPV1, G protein–coupled receptor 55, and adenosine transport proteins (111, 112, 113). CBD inhibits ion currents from many structurally diverse voltage-gated ion channels at similar micromolar concentrations, and with a high degree of cooperativity, suggesting that CBD acts indirectly on ion channels through perturbation of membrane structure (20). Moving forward, it will be important to determine the role of lipid order and cholesterol orientation in the mediation of CBD-induced effects in models of generalized seizures.

Our study suggests that not all CBD effects on cells are therapeutically beneficial and that high-dose use of CBD may lead to cholesterol-dependent side effects in certain cell types that rely on high levels of cholesterol synthesis or import. We demonstrated that CBD-driven apoptosis is heavily dependent on the cholesterol status of cells (Figs. 5*B*, 5*C*, S5, *A* and *B*). As a large fraction of cellular cholesterol in humans is synthesized in hepatocytes, we predict that many of the side effects of heavy CBD consumption may occur in the liver. Adverse events in CBD clinical trials include elevated liver aminotransferase levels (114), a hallmark of liver injury (115), which suggests that CBD may elevate the risk of hepatoxicity. Our data point to the importance of testing whether CBD use may interact adversely with certain dietary behaviors that elevate blood cholesterol, as the combination of cholesterol/hydroxycholesterol and CBD is toxic to a cell line derived from skin (HaCaT cells), brain (SK-N-BE(2) cells), and kidney (HEK293T cells) (Figs. 5*D*, 5*E*, S5*B*). Furthermore, our results that CBD disrupted cholesterol trafficking through lysosomes, when in combination with U18666A, raises the question of whether CBD use might increase the risk of toxicity in patients with Niemann–Pick disease type C, which harbor mutations in NPC1, the target of U18666A (116).

## Resource Availability

All unique/stable reagents generated in this study are available from Michael H.B. Stowell (michael.stowell@colorado.edu), with a completed Materials Transfer Agreement.

## Data Availability

Proteomics and phosphoproteomics raw data are available at the MassIVE repository ID MSV000085479 accessible at https://doi.org/https://doi.org/10.25345/C5571V. A zip file containing MaxQuant output files and raw files can be found at f.MSV000085479/updates/2022-03-18_wold_cub_72df2146/other/TP17_SKNBe2_CBD_phospho.zip, and the mqpar.xml file for reanalysis of the phosphoproteome raw files with MaxQuant can be found at f.MSV000085479/updates/2022-05-03_wold_cub_23a4d923/search/mqpar.xml. Please note that to use the MaxQuant viewer, the mqpar.xml file must be copied to the same directory as the raw files, associated index files, and combined folder.

Source data for RNA-Seq experiments is accessible at Gene Expression Omnibus with the identifier GSE151512 at https://www.ncbi.nlm.nih.gov/geo/query/acc.cgi?acc=GSE151512.

The code generated during this study is available at GitHub, https://doi.org/10.5281/zenodo.3861043, URL: https://github.com/CUOldLab/cbd-manuscript-code-guardse-2020.

## Supplemental data

This article contains supplemental data.

## Conflict of interest

D. A. C., X. L., and W. M. O. are patent holders of PCT WO2019246632A1, and, D. A. C., X. L., W. M. O., and E. B. are patent holders of PCT WO2019118837A1. Both patents are related to this work. Unrelated to the contents of this article, A. D. A. and T. N. are founders of Omix Technologies, Inc; D. A. C. is the founder of BioLoomics, Inc; and R.S. is the founder of Sievers Infinity LLC. All the other authors declare no competing interests.

## Supplemental Data

Supplemental Table 1_withCaption

supp-2

supp-3

supp-4

supp-5

supp-6

supp-7

supp-8

SubcellularProteome_proteinGroups

Combined Supplement and Legends_UpdatedTableS1caption

## References

[1] Devinsky O., Cross J.H., Laux L., Marsh E., Miller I., Nabbout R. (2017). Trial of cannabidiol for drug-resistant seizures in the dravet syndrome. N. Engl. J. Med..

[2] Miller I., Scheffer I.E., Gunning B., Sanchez-Carpintero R., Gil-Nagel A., Perry M.S. (2020). Dose-ranging effect of adjunctive oral cannabidiol vs placebo on convulsive seizure frequency in Dravet syndrome: a randomized clinical trial. JAMA Neurol..

[3] Thiele E.A., Marsh E.D., French J.A., Mazurkiewicz-Beldzinska M., Benbadis S.R., Joshi C. (2018). Cannabidiol in patients with seizures associated with lennox-gastaut syndrome (GWPCARE4): a randomised, double-blind, placebo-controlled phase 3 trial. Lancet.

[4] McPartland J.M., Duncan M., Di Marzo V., Pertwee R.G. (2015). Are cannabidiol and Δ(9) -tetrahydrocannabivarin negative modulators of the endocannabinoid system? A systematic review. Br. J. Pharmacol..

[5] Iffland K., Grotenhermen F. (2017). An update on safety and side effects of cannabidiol: a review of clinical data and relevant animal studies. Cannabis Cannabinoid Res..

[6] De Filippis D., Esposito G., Cirillo C., Cipriano M., De Winter B.Y., Scuderi C. (2011). Cannabidiol reduces intestinal inflammation through the control of neuroimmune axis. PLoS One.

[7] Hinz B., Ramer R. (2019). Anti-tumour actions of cannabinoids. Br. J. Pharmacol..

[8] Kenyon J., Liu W., Dalgleish A. (2018). Report of objective clinical responses of cancer patients to pharmaceutical-grade synthetic cannabidiol. Anticancer Res..

[9] Massi P., Solinas M., Cinquina V., Parolaro D. (2013). Cannabidiol as potential anticancer drug. Br. J. Clin. Pharmacol..

[10] McAllister S.D., Soroceanu L., Desprez P.-Y. (2015). The antitumor activity of plant-derived non-psychoactive cannabinoids. J. Neuroimmune Pharmacol..

[11] Esposito G., Scuderi C., Valenza M., Togna G.I., Latina V., De Filippis D. (2011). Cannabidiol reduces Aβ-induced neuroinflammation and promotes hippocampal neurogenesis through PPARγ involvement. PLoS One.

[12] Pan H., Mukhopadhyay P., Rajesh M., Patel V., Mukhopadhyay B., Gao B. (2009). Cannabidiol attenuates cisplatin-induced nephrotoxicity by decreasing oxidative/nitrosative stress, inflammation, and cell death. J. Pharmacol. Exp. Ther..

[13] Rajesh M., Mukhopadhyay P., Bátkai S., Patel V., Saito K., Matsumoto S. (2010). Cannabidiol attenuates cardiac dysfunction, oxidative stress, fibrosis, and inflammatory and cell death signaling pathways in diabetic cardiomyopathy. J. Am. Coll. Cardiol..

[14] Devinsky O., Cilio M.R., Cross H., Fernandez-Ruiz J., French J., Hill C. (2014). Cannabidiol: pharmacology and potential therapeutic role in epilepsy and other neuropsychiatric disorders. Epilepsia.

[15] Devinsky O., Patel A.D., Thiele E.A., Wong M.H., Appleton R., Harden C.L. (2018). Randomized, dose-ranging safety trial of cannabidiol in Dravet syndrome. Neurology.

[16] Ahrens J., Demir R., Leuwer M., de la Roche J., Krampfl K., Foadi N. (2009). The nonpsychotropic cannabinoid cannabidiol modulates and directly activates alpha-1 and alpha-1-Beta glycine receptor function. Pharmacology.

[17] Ibeas Bih C., Chen T., Nunn A.V., Bazelot M., Dallas M., Whalley B.J. (2015). Molecular targets of cannabidiol in neurological disorders. Neurotherapeutics.

[18] Lauckner J.E., Jensen J.B., Chen H.Y., Lu H.C., Hille B., Mackie K. (2008). GPR55 is a cannabinoid receptor that increases intracellular calcium and inhibits M current. Proc. Natl. Acad. Sci. U. S. A..

[19] Whyte L.S., Ryberg E., Sims N.A., Ridge S.A., Mackie K., Greasley P.J. (2009). The putative cannabinoid receptor GPR55 affects osteoclast function *in vitro* and bone mass *in vivo*. Proc. Natl. Acad. Sci. U. S. A..

[20] Ghovanloo M.-R., Shuart N.G., Mezeyova J., Dean R.A., Ruben P.C., Goodchild S.J. (2018). Inhibitory effects of cannabidiol on voltage-dependent sodium currents. J. Biol. Chem..

[21] Dravet C. (2011). The core Dravet syndrome phenotype. Epilepsia.

[22] Ross H.R., Napier I., Connor M. (2008). Inhibition of recombinant human T-type calcium channels by Delta9-tetrahydrocannabinol and cannabidiol. J. Biol. Chem..

[23] Rimmerman N., Ben-Hail D., Porat Z., Juknat A., Kozela E., Daniels M.P. (2013). Direct modulation of the outer mitochondrial membrane channel, voltage-dependent anion channel 1 (VDAC1) by cannabidiol: a novel mechanism for cannabinoid-induced cell death. Cell Death Dis..

[24] Gray R.A., Whalley B.J. (2020). The proposed mechanism of action of CBD in epilepsy. Epileptic Disord..

[25] Lundbaek J.A., Birn P., Tape S.E., Toombes G.E., Søgaard R., Koeppe R.E. (2005). Capsaicin regulates voltage-dependent sodium channels by altering lipid bilayer elasticity. Mol. Pharmacol..

[26] Lundbæk J.A., Birn P., Girshman J., Hansen A.J., Andersen O.S. (1996). Membrane stiffness and channel function. Biochemistry.

[27] Watkins A.R. (2019). Cannabinoid interactions with ion channels and receptors. Channels (Austin).

[28] Rimmerman N., Juknat A., Kozela E., Levy R., Bradshaw H.B., Vogel Z. (2011). The non-psychoactive plant cannabinoid, cannabidiol affects cholesterol metabolism-related genes in microglial cells. Cell Mol. Neurobiol..

[29] Silvestri C., Paris D., Martella A., Melck D., Guadagnino I., Cawthorne M. (2015). Two non-psychoactive cannabinoids reduce intracellular lipid levels and inhibit hepatosteatosis. J. Hepatol..

[30] Wang Y., Mukhopadhyay P., Cao Z., Wang H., Feng D., Haskó G. (2017). Cannabidiol attenuates alcohol-induced liver steatosis, metabolic dysregulation, inflammation and neutrophil-mediated injury. Sci. Rep..

[31] Blum B.C., Mousavi F., Emili A. (2018). Single-platform ‘multi-omic’ profiling: Unified mass spectrometry and computational workflows for integrative proteomics–metabolomics analysis. Mol. Omics.

[32] Hafner M., Mills C.E., Subramanian K., Chen C., Chung M., Boswell S.A. (2019). Multiomics profiling establishes the polypharmacology of FDA-approved CDK4/6 inhibitors and the potential for differential clinical activity. Cell Chem. Biol..

[33] Norris J.L., Farrow M.A., Gutierrez D.B., Palmer L.D., Muszynski N., Sherrod S.D. (2017). Integrated, high-throughput, multiomics platform enables data-driven construction of cellular responses and reveals global drug mechanisms of action. J. Proteome Res..

[34] Chapnick D.A., Bunker E., Liu X. (2015). A biosensor for the activity of the ‘sheddase’ TACE (ADAM17) reveals novel and cell type-specific mechanisms of TACE activation. Sci. Signal..

[35] Chapnick D.A., Liu X. (2014). Leader cell positioning drives wound-directed collective migration in TGFβ-stimulated epithelial sheets. Mol. Biol. Cell.

[36] Trapnell C., Williams B.A., Pertea G., Mortazavi A., Kwan G., van Baren M.J. (2010). Transcript assembly and quantification by RNA-Seq reveals unannotated transcripts and isoform switching during cell differentiation. Nat. Biotechnol..

[37] Jow H., Boys R.J., Wilkinson D.J. (2014). Bayesian identification of protein differential expression in multi-group isobaric labelled mass spectrometry data. Stat. Appl. Genet. Mol. Biol..

[38] Meyer-Arendt K., Old W.M., Houel S., Renganathan K., Eichelberger B., Resing K.A. (2011). IsoformResolver: a peptide-centric algorithm for protein inference. J. Proteome Res..

[39] Denwood M. (2016). runjags: An R package providing interface utilities, model templates, parallel computing methods and additional distributions for MCMC models in JAGS. J Stat Softw.

[40] Gelman A., Rubin D.B. (1992). Inference from iterative simulation using multiple sequences. Stat. Sci..

[41] Leek J.T., Monsen E., Dabney A.R., Storey J.D. (2006). Edge: extraction and analysis of differential gene expression. Bioinformatics.

[42] Benjamini Y., Hochberg Y. (1995). Controlling the false discovery rate: a practical and powerful approach to multiple testing. J. R. Stat. Soc. Ser. B (Methodological).

[43] Kuleshov M.V., Jones M.R., Rouillard A.D., Fernandez N.F., Duan Q., Wang Z. (2016). Enrichr: a comprehensive gene set enrichment analysis web server 2016 update. Nucl. Acids Res..

[44] Wiśniewski J.R. (2016). Quantitative evaluation of filter aided sample preparation (FASP) and multienzyme digestion FASP protocols. Anal. Chem..

[45] Nemkov T., Hansen K.C., D’Alessandro A. (2017). A three-minute method for high-throughput quantitative metabolomics and quantitative tracing experiments of central carbon and nitrogen pathways. Rapid Commun. Mass Spectrom..

[46] Nemkov T., Reisz J.A., Gehrke S., Hansen K.C., D’Alessandro A. (2019).

[47] Clasquin M.F., Melamud E., Rabinowitz J.D. (2012). LC-MS data processing with MAVEN: a metabolomic analysis and visualization engine. Curr. Protoc. Bioinformatics.

[48] Chong J., Soufan O., Li C., Caraus I., Li S., Bourque G. (2018). MetaboAnalyst 4.0: towards more transparent and integrative metabolomics analysis. Nucl. Acids Res..

[49] Reisz J.A., Zheng C., D’Alessandro A., Nemkov T. (2019). Untargeted and semi-targeted lipid analysis of biological samples using mass spectrometry-based metabolomics. Methods Mol. Biol..

[50] Widenmaier S.B., Snyder N.A., Nguyen T.B., Arduini A., Lee G.Y., Arruda A.P. (2017). NRF1 is an ER membrane sensor that is central to cholesterol homeostasis. Cell.

[51] Chapnick D.A., Bunker E., Liu X., Old W.M. (2019). Temporal metabolite, ion, and enzyme activity profiling using fluorescence microscopy and genetically encoded biosensors. Methods Mol. Biol..

[52] Hawley S.A., Boudeau J., Reid J.L., Mustard K.J., Udd L., Mäkelä T.P. (2003). Complexes between the LKB1 tumor suppressor, STRAD alpha/beta and MO25 alpha/beta are upstream kinases in the AMP-activated protein kinase cascade. J. Biol..

[53] Hawley S.A., Pan D.A., Mustard K.J., Ross L., Bain J., Edelman A.M. (2005). Calmodulin-dependent protein kinase kinase-β is an alternative upstream kinase for AMP-activated protein kinase. Cell Metab..

[54] Shaw R.J., Kosmatka M., Bardeesy N., Hurley R.L., Witters L.A., DePinho R.A. (2004). The tumor suppressor LKB1 kinase directly activates AMP-activated kinase and regulates apoptosis in response to energy stress. Proc. Natl. Acad. Sci. U. S. A..

[55] Woods A., Dickerson K., Heath R., Hong S.P., Momcilovic M., Johnstone S.R. (2005). Ca2+/calmodulin-dependent protein kinase kinase-beta acts upstream of AMP-activated protein kinase in mammalian cells. Cell Metab..

[56] Ong S.-E., Blagoev B., Kratchmarova I., Kristensen D.B., Steen H., Pandey A. (2002). Stable isotope labeling by amino acids in cell culture, SILAC, as a simple and accurate approach to expression proteomics. Mol. Cell Proteomics.

[57] Schaffer B.E., Levin R.S., Hertz N.T., Maures T.J., Schoof M.L., Hollstein P.E. (2015). Identification of AMPK phosphorylation sites reveals a network of proteins involved in cell invasion and facilitates large-scale substrate prediction. Cell Metab..

[58] Dale S., Wilson W.A., Edelman A.M., Hardie D.G. (1995). Similar substrate recognition motifs for mammalian AMP-activated protein kinase, higher plant HMG-CoA reductase kinase-A, yeast SNF1, and mammalian calmodulin-dependent protein kinase I. FEBS Lett..

[59] Gwinn D.M., Shackelford D.B., Egan D.F., Mihaylova M.M., Mery A., Vasquez D.S. (2008). AMPK phosphorylation of raptor mediates a metabolic checkpoint. Mol. Cell.

[60] Li X., Wang L., Zhou X.E., Ke J., de Waal P.W., Gu X. (2015). Structural basis of AMPK regulation by adenine nucleotides and glycogen. Cell Res..

[61] Carlson C.A., Kim K.-H. (1973). Regulation of hepatic acetyl coenzyme A carboxylase by phosphorylation and dephosphorylation. J. Biol. Chem..

[62] Munday M.R. (2002). Regulation of mammalian acetyl-CoA carboxylase. Biochem. Soc. Trans..

[63] Fediuc S., Gaidhu M.P., Ceddia R.B. (2006). Regulation of AMP-activated protein kinase and acetyl-CoA carboxylase phosphorylation by palmitate in skeletal muscle cells. J. Lipid Res..

[64] McFadden J.W., Corl B.A. (2009). Activation of AMP-activated protein kinase (AMPK) inhibits fatty acid synthesis in bovine mammary epithelial cells. Biochem. Biophys. Res. Commun..

[65] Ryazanov A.G., Shestakova E.A., Natapov P.G. (1988). Phosphorylation of elongation factor 2 by EF-2 kinase affects rate of translation. Nature.

[66] BeltrandelRio H., Wilson J.E. (1992). Coordinated regulation of cerebral glycolytic and oxidative metabolism, mediated by mitochondrially bound hexokinase dependent on intramitochondrially generated ATP. Arch. Biochem. Biophys..

[67] Crane R.K., Sols A. (1953). The association of hexokinase with particulate fractions of brain and other tissue homogenates. J. Biol. Chem..

[68] Saraiva L.M., Seixas da Silva G.S., Galina A., da-Silva W.S., Klein W.L., Ferreira S.T. (2010). Amyloid-β triggers the release of neuronal hexokinase 1 from mitochondria. PLoS One.

[69] Alharris E., Singh N.P., Nagarkatti P.S., Nagarkatti M. (2019). Role of miRNA in the regulation of cannabidiol-mediated apoptosis in neuroblastoma cells. Oncotarget.

[70] Krämer A., Green J., Pollard J., Tugendreich S. (2014). Causal analysis approaches in ingenuity pathway analysis. Bioinformatics.

[71] Blais J.D., Filipenko V., Bi M., Harding H.P., Ron D., Koumenis C. (2004). Activating transcription factor 4 is translationally regulated by hypoxic stress. Mol. Cell Biol..

[72] Dasari A., Bartholomew J.N., Volonte D., Galbiati F. (2006). Oxidative stress induces premature senescence by stimulating caveolin-1 gene transcription through p38 mitogen-activated protein kinase/sp1–mediated activation of two GC-rich promoter Elements. Cancer Res..

[73] Venugopal R., Jaiswal A.K. (1998). Nrf2 and Nrf1 in association with Jun proteins regulate antioxidant response element-mediated expression and coordinated induction of genes encoding detoxifying enzymes. Oncogene.

[74] Wang G.L., Semenza G.L. (1993). General involvement of hypoxia-inducible factor 1 in transcriptional response to hypoxia. Proc. Natl. Acad. Sci. U. S. A..

[75] Supek F., Bošnjak M., Škunca N., Šmuc T. (2011). REVIGO summarizes and Visualizes long lists of gene ontology terms. PLoS One.

[76] De Felice B., Santillo M., Serù R., Damiano S., Matrone G., Wilson R.R. (2004). Modulation of 3-hydroxy-3-methylglutaryl-CoA reductase gene expression by CuZn superoxide dismutase in human fibroblasts and HepG2 cells. Gene Expr..

[77] Brown M.S., Ho Y.K., Goldstein J.L. (1980). The cholesteryl ester cycle in macrophage foam cells. Continual hydrolysis and re-esterification of cytoplasmic cholesteryl esters. J. Biol. Chem..

[78] Chang T.Y., Li B.L., Chang C.C.Y., Urano Y. (2009). Acyl-coenzyme A:cholesterol acyltransferases. Am. J. Physiol. - Endocrinol. Metab..

[79] Zhao Y.-F., Wang L., Lee S., Sun Q., Tuo Y., Wang Y. (2010). Cholesterol induces mitochondrial dysfunction and apoptosis in mouse pancreatic beta-cell line MIN6 cells. Endocr.

[80] Chang T.-Y., Reid P.C., Sugii S., Ohgami N., Cruz J.C., Chang C.C. (2005). Niemann-pick type C disease and intracellular cholesterol trafficking. J. Biol. Chem..

[81] Bang B., Gniadecki R., Gajkowska B. (2005). Disruption of lipid rafts causes apoptotic cell death in HaCaT keratinocytes. Exp. Dermatol..

[82] Demais V., Barthélémy A., Perraut M., Ungerer N., Keime C., Reibel S. (2016). Reversal of pathologic lipid accumulation in NPC1-deficient neurons by drug-promoted release of LAMP1-coated lamellar inclusions. J. Neurosci..

[83] Höglinger D., Burgoyne T., Sanchez-Heras E., Hartwig P., Colaco A., Newton J. (2019). NPC1 regulates ER contacts with endocytic organelles to mediate cholesterol egress. Nat. Commun..

[84] Cheng D., Chang C.C., Qu X., Chang T.Y. (1995). Activation of acyl-coenzyme A:cholesterol acyltransferase by cholesterol or by oxysterol in a cell-free system. J. Biol. Chem..

[85] Lange Y., Ye J., Rigney M., Steck T.L. (1999). Regulation of endoplasmic reticulum cholesterol by plasma membrane cholesterol. J. Lipid Res..

[86] Ahn K., Sampson N.S. (2004). Cholesterol oxidase senses subtle changes in lipid bilayer structure. Biochemistry.

[87] Ortegren U., Karlsson M., Blazic N., Blomqvist M., Nystrom F.H., Gustavsson J. (2004). Lipids and glycosphingolipids in caveolae and surrounding plasma membrane of primary rat adipocytes. Eur. J. Biochem..

[88] Smart E.J., Ying Y.S., Conrad P.A., Anderson R.G. (1994). Caveolin moves from caveolae to the Golgi apparatus in response to cholesterol oxidation. J. Cell Biol..

[89] Ferreri C. (2005). Life—as a matter of fat: the emerging science of Lipidomics.By Ole G. Mouritsen. Chembiochem.

[90] Lindblom G., Orädd G. (2009). Lipid lateral diffusion and membrane heterogeneity. Biochim. Biophys. Acta.

[91] George K.S., Wu S. (2012). Lipid raft: a floating island of death or survival. Toxicol. Appl. Pharmacol..

[92] Wassall S.R., Leng X., Canner S.W., Pennington E.R., Kinnun J.J., Cavazos A.T. (2018). Docosahexaenoic acid regulates the formation of lipid rafts: a unified view from experiment and simulation. Biochim. Biophys. Acta Biomembr..

[93] Geng L., Zhou W., Liu B., Wang X., Chen B. (2018). DHA induces apoptosis of human malignant breast cancer tissues by the TLR-4/PPAR-α pathways. Oncol. Lett..

[94] Serini S., Trombino S., Oliva F., Piccioni E., Monego G., Resci F. (2008). Docosahexaenoic acid induces apoptosis in lung cancer cells by increasing MKP-1 and down-regulating p-ERK1/2 and p-p38 expression. Apoptosis.

[95] Shin S., Jing K., Jeong S., Kim N., Song K.S., Heo J.Y. (2013). The omega-3 polyunsaturated fatty acid DHA induces simultaneous apoptosis and autophagy *via* mitochondrial ROS-mediated Akt-mTOR signaling in prostate cancer cells expressing mutant p53. Biomed. Res. Int..

[96] Sun S.-N., Jia W.D., Chen H., Ma J.L., Ge Y.S., Yu J.H. (2013). Docosahexaenoic acid (DHA) induces apoptosis in human hepatocellular carcinoma cells. Int. J. Clin. Exp. Pathol..

[97] Schnitzer J.E., Oh P., Pinney E., Allard J. (1994). Filipin-sensitive caveolae-mediated transport in endothelium: Reduced transcytosis, scavenger endocytosis, and capillary permeability of select macromolecules. J. Cell Biol..

[98] Phan B.A.P., Dayspring T.D., Toth P.P. (2012). Ezetimibe therapy: mechanism of action and clinical update. Vasc. Health Risk Manag..

[99] Carreras-Sureda A., Pihán P., Hetz C. (2018). Calcium signaling at the endoplasmic reticulum: Fine-tuning stress responses. Cell Calcium.

[100] Brown M.S., Goldstein J.L. (1997). The SREBP pathway: Regulation of cholesterol metabolism by proteolysis of a membrane-bound transcription factor. Cell.

[101] Besprozvannaya M., Dickson E., Li H., Ginburg K.S., Bers D.M., Auwerx J. (2018). GRAM domain proteins specialize functionally distinct ER-PM contact sites in human cells. Elife.

[102] Naito T., Ercan B., Krshnan L., Triebl A., Koh D.H.Z., Wei F.Y. (2019). Movement of accessible plasma membrane cholesterol by the GRAMD1 lipid transfer protein complex. Elife.

[103] Das A., Brown M.S., Anderson D.D., Goldstein J.L., Radhakrishnan A. (2014). Three pools of plasma membrane cholesterol and their relation to cholesterol homeostasis. Elife.

[104] Lange Y., Tabei S.M.A., Ye J., Steck T.L. (2013). Stability and stoichiometry of bilayer phospholipid–cholesterol complexes: relationship to cellular sterol distribution and homeostasis. Biochemistry.

[105] Sokolov A., Radhakrishnan A. (2010). Accessibility of cholesterol in endoplasmic reticulum membranes and activation of SREBP-2 switch abruptly at a common cholesterol threshold. J. Biol. Chem..

[106] Gianfrancesco M.A., Paquot N., Piette J., Legrand-Poels S. (2018). Lipid bilayer stress in obesity-linked inflammatory and metabolic disorders. Biochem. Pharmacol..

[107] Hsu J.-L., Leu W.J., Hsu L.C., Liu S.P., Zhong N.S., Guh J.H. (2018). Para-toluenesulfonamide induces anti-tumor activity through akt-dependent and -independent mTOR/p70S6K pathway: roles of lipid raft and cholesterol contents. Front. Pharmacol..

[108] Mollinedo F., Gajate C. (2015). Lipid rafts as major platforms for signaling regulation in cancer. Adv. Biol. Regul..

[109] Pirmoradi L., Seyfizadeh N., Ghavami S., Zeki A.A., Shojaei S. (2019). Targeting cholesterol metabolism in glioblastoma: A new therapeutic approach in cancer therapy. J. Investig. Med..

[110] Staneva G., Puff N., Stanimirov S., Tochev T., Angelova M.I., Seigneuret M. (2018). The Alzheimer’s disease amyloid-β peptide affects the size-dynamics of raft-mimicking Lo domains in GM1-containing lipid bilayers. Soft Matter.

[111] Bisogno T., Hanus L., De Petrocellis L., Tchilibon S., Ponde D.E., Brandi I. (2001). Molecular targets for cannabidiol and its synthetic analogues: effect on vanilloid VR1 receptors and on the cellular uptake and enzymatic hydrolysis of anandamide. Br. J. Pharmacol..

[112] Liou G.I., Auchampach J.A., Hillard C.J., Zhu G., Yousufzai B., Mian S. (2008). Mediation of cannabidiol anti-inflammation in the retina by equilibrative nucleoside transporter and A2A adenosine receptor. Invest. Ophthalmol. Vis. Sci..

[113] Ryberg E., Larsson N., Sjögren S., Hjorth S., Hermansson N.O., Leonova J. (2007). The orphan receptor GPR55 is a novel cannabinoid receptor. Br. J. Pharmacol..

[114] Gaston T.E., Bebin E.M., Cutter G.R., Liu Y., Szaflarski J.P., UAB CBD Program (2017). Interactions between cannabidiol and commonly used antiepileptic drugs. Epilepsia.

[115] Giannini E.G., Testa R., Savarino V. (2005). Liver enzyme alteration: A guide for clinicians. CMAJ.

[116] Lu F., Liang Q., Abi-Mosleh L., Das A., De Brabander J.K., Goldstein J.L. (2015). Identification of NPC1 as the target of U18666A, an inhibitor of lysosomal cholesterol export and Ebola infection. Elife.

