# Supplementary material for: Multiomic Analysis Reveals Disruption of Cholesterol Homeostasis by Cannabidiol in Human Cell Lines
Source: Mol Cell Proteomics. 2022 Jun 24;21(10):100262. doi: 10.1016/j.mcpro.2022.100262 (PMC9525918; doi:10.1016/j.mcpro.2022.100262)
Supplement: Supplemental Table 1_withCaption [file mmc1.pdf]

Table S1. Biosensors used for profiling the EC50 Effects of CBD, related to Figure 1 and Supplemental Figure 1

Targeted Analyte	Biosensor Gene Name	Original Literature Source
Adam17 Protease Activity	TSEN	Chapnick DA, Bunker E, Liu X (2015) A biosensor for the activity of the "sheddase" TACE (ADAM17) reveals novel and cell type-specific mechanisms of TACE activation. Science signaling 8 (365)
AMP Kinase Activity	AMPKAR	Tsou P, Zheng B, Hsu CH, Sasaki AT, Cantley LC (2011) A fluorescent reporter of AMPK activity and cellular energy stress. Cell metabolism 13 (4):476-486
ATP Abundance	ATEAM	Imamura H, Nhat KP, Togawa H, Saito K, Iino R, Kato-Yamada Y, Nagai T, Noji H (2009) Visualization of ATP levels inside single living cells with fluorescence resonance energy transfer-based genetically encoded indicators. Proceedings of the National Academy of Sciences of the United States of America 106 (37):15651-15656
Cytosolic Ca^{2+} Abundance	D3-cpv	Ravier MA, Cheng-Xue R, Palmer AE, Henquin JC, Gilon P (2010) Subplasmalemmal Ca(2+) measurements in mouse pancreatic beta cells support the existence of an amplifying effect of glucose on insulin secretion. Diabetologia 53 (9):1947-1957
Cytosolic ERK Activity	EKAR-NES	Komatsu N, Aoki K, Yamada M, Yukinaga H, Fujita Y, Kamioka Y, Matsuda M (2011) Development of an optimized backbone of FRET biosensors for kinases and GTPases. Molecular biology of the cell 22 (23):4647-4656
ER Ca^{2+} Abundance	D1ER	Palmer AE, Jin C, Reed JC, Tsien RY (2004) Bcl-2-mediated alterations in endoplasmic reticulum Ca2+ analyzed with an improved genetically encoded fluorescent sensor. Proceedings of the National Academy of Sciences of the United States of America 101 (50):17404-17409
Glucose Abundance	FLIPglu-30uDelta13V	Takanaga H, Frommer WB (2010) Facilitative plasma membrane transporters function during ER transit. FASEB journal : official publication of the Federation of American Societies for Experimental Biology 24 (8):2849-2858

Glutamine Abundance	FLIPQTV3.0 8m	Gruenwald K, Holland JT, Stromberg V, Ahmad A, Watcharakichkorn D, Okumoto S (2012) Visualization of glutamine transporter activities in living cells using genetically encoded glutamine sensors. PloS one 7 (6)
Lactate Abundance	Laconic	San Martin A, Ceballo S, Ruminot I, Lerchundi R, Frommer WB, Barros LF (2013) A genetically encoded FRET lactate sensor and its use to detect the Warburg effect in single cancer cells. PloS one 8 (2)
mTOR Kinase Activity	TORCAR	Zhou X, Clister TL, Lowry PR, Seldin MM, Wong GW, Zhang J (2015) Dynamic Visualization of mTORC1 Activity in Living Cells. Cell reports
PKD Kinase Activity	DKAR	Kunkel MT, Toker A, Tsien RY, Newton AC (2007) Calcium-dependent regulation of protein kinase D revealed by a genetically encoded kinase activity reporter. The Journal of biological chemistry 282 (9):6733-6742
Plasma Membrane Electrostatic Potential (Charge)	MCS+	Ma Y, Yamamoto Y, Nicovich PR, Goyette J, Rossy J, Gooding JJ, Gaus K (2017) A FRET sensor enables quantitative measurements of membrane charges in live cells. Nature biotechnology 35 (4):363-370
Plasma Membrane Potential	VSFP-CR	Lam AJ, St-Pierre F, Gong Y, Marshall JD, Cranfill PJ, Baird MA, McKeown MR, Wiedenmann J, Davidson MW, Schnitzer MJ, Tsien RY, Lin MZ (2012) Improving FRET dynamic range with bright green and red fluorescent proteins. Nature methods 9 (10):1005-1012
Pyruvate Abundance	Pyronic	San Martin A, Ceballo S, Baeza-Lehnert F, Lerchundi R, Valdebenito R, Contreras-Baeza Y, Alegria K, Barros LF (2014) Imaging mitochondrial flux in single cells with a FRET sensor for pyruvate. PloS one 9 (1)
