# Supplementary material for: Multiomic Analysis Reveals Disruption of Cholesterol Homeostasis by Cannabidiol in Human Cell Lines
Source: Mol Cell Proteomics. 2022 Jun 24;21(10):100262. doi: 10.1016/j.mcpro.2022.100262 (PMC9525918; doi:10.1016/j.mcpro.2022.100262)
Supplement: Combined Supplement and Legends_UpdatedTableS1caption [file mmc10.pdf]

Supplemental Information Legends:

Table S1. Biosensors used for profiling the EC50 Effects of CBD, related to Figure 1 and Supplemental Figure 1

The name of each genetically encoded biosensor gene used to profile diverse activities in cell lines is displayed along with its target analyte and literature source.

Supplemental Figure 1. FRET-based biosensor screening reveals dose dependent molecular responses to CBD

(A) Genetically encoded FRET biosensors for diverse biochemical pathways were delivered to both SK-N-BE(2) and HaCaT Cells. Heatmaps display normalized FRET ratio over 20 minute time intervals from 0.343 μM to 100 μM CBD treatment.

Supplemental Figure 2. CBD-dependent phosphorylation changes are enriched in AMPK substrate motifs

(A) Volcano plot of phosphorylation sites identified by LC/MS 10 minutes post treatment with 20 μM CBD. 5 proteins were identified with adjusted $p < 0.05$ and $FC > |0.5|$ by 10 minutes (text in white box / red dot label) **(B)** BioPlanet Pathway enrichment of significantly changing phosphorylation sites at 1 and 3 hours post CBD treatment. * adjusted $p < 0.05$; ** adjusted $p < 0.01$ **(C-D)** Motif enrichment tables for significantly changing phosphorylated sites against global background at 1 and 3 hours post treatment with 20 μM CBD. **(E)** Abundance of *de novo* synthesized and elongated fatty acids are shown for $^{13}C_2$-butanoic acid (C4) and $^{13}C_2$-octadecenoic acid (C18:1) in both vehicle-treated (blue) and CBD-treated (20 μM) cells (red) Lines represent median values and shaded areas covered standard error of the median **(F)** The relative abundance of acylcarnitines (AC) are shown as a heat map with abundance plotted by Z-score according to a color gradient from blue to red for lowest to highest abundance. The acyl-chain length is described in parentheses. **(G)** The relative levels of $^{13}C_2$ incorporation into lactate and TCA cycle intermediates in both vehicle-treated (blue) and CBD-treated cells (red).

Supplemental Figure 3. pH-dependent subcellular fractionation generates compositionally distinct proteome fractions

(A) Principal component analysis (PCA) of proteome fractions in a CBD time course based on bioconductor EDGE analysis of 5 replicate pairs as described in the STAR METHODS **(B)** PCA of proteome fractions and whole cell lysate in untreated conditions. Each dot represents a biological replicate. **(C-E)** Cellular component enrichment analysis of proteins identified in each of the proteome fractions. Significantly enriched gene ontologies from each fraction are plotted by log of the false discovery rate (FDR) versus enrichment ratio. Dot color is scaled on the number of proteins found within each enrichment. **(F)** Differential expression for HK1 between membrane and nuclear fractions over time. **(G)** Relative abundance of Hexose phosphate (representing a pool of sugar phosphates largely including glucose 6-phosphate) and reduced glutathione is shown. Lines represent median values and shaded areas covered standard error of the median. **(H)** Upstream regulator analysis of differentially expressed transcripts

measured by RNAseq in CBD treated cells. Number of genes enriched at each timepoint are displayed on the right.

Supplemental Figure 4. CBD treatment results in increased flux of biosynthetic precursors by 24 hours

(A-B) Plots of ^{13}C labeled and total + labeled abundances of cholesterol biosynthesis metabolites measured in methanol extracts from SK-N-BE(2) cells treated with vehicle or 20 µM CBD. **(C)** Fluorometric measurement of total cellular cholesterol from methanol extracts of SK-N-BE(2) cells using the Amplex Red Cholesterol Assay Kit. **(D)** Lipid head groups detected by LC/MS/MS are displayed by peak area. *: $p < 0.05$, student's T-Test) **(E)** Volcano plot of all lipid species detected by LC/MS/MS from lipid extracts of SK-N-BE(2) cells treated with 20 µM CBD for 24 hrs. Red line: $p < 0.05$ with FDR correction. Significant species are labeled. **(F)** Live cell confocal microscopy of SK-N-BE(2) cells exposed to CBD (20 µM) stained with fluorescent cholesterol and Nile red. Scale bar 5 µm. Quantification of lipid droplet count per cell and lipid droplet size from confocal experiments is displayed (n=2)

Supplemental Figure 5. CBD-dependent apoptosis is cholesterol dependent across cell lines

(A) HACAT cells were assessed for apoptosis at 24 hours using live cell microscopy using a resazurin based fluorometric cell viability stain. Cells were treated with 10 µM atorvastatin and exposed to increasing doses of CBD. **(B)** HACAT cells were treated with 20 µM CBD and exposure to increasing doses of 25-OH cholesterol. **(C)** SK-N-BE(2) and HEK293T cells were treated with different combinations of 20 µM CBD, 10 µM U18666A, 5µM VULM and 15µg/ ml 25- OH Cholesterol and were measured for apoptosis rates over time.

Supplemental Figure 6. CBD alters cholesterol orientation in complex membranes

(A) Synthetic small unilamellar vesicles (SUVs) free of cholesterol and composed of 100% Phosphatidylcholine were analyzed with a fluorogenic cholesterol oxidase reaction to determine the effect of CBD on initial reaction rate in the presence and absence of cholesterol oxidase enzyme. **(B)** 1µg/ reaction of free 25-OH cholesterol was used in the absence of SUVs in a cholesterol oxidase fluorogenic assay to measure reaction rate with non-membrane resident cholesterol. **(C)** ER derived vesicles were used as a source of cholesterol for similar experiments as displayed in suppl 6B. **(D-E)** SK-N-BE(2) and HEK293T cells were treated with increasing amounts of DHA and evaluated for apoptosis. DHA (75 µM) was then evaluated for rescue by either low dose CBD (6.25 µM) or MBCD (300 nM). **(F)** Fluorescent measurement of propidium iodide uptake in live cells treated with 20 µM CBD and a dose curve of Filipin at 24 hours. CBD increased membrane permeability to propidium iodide.

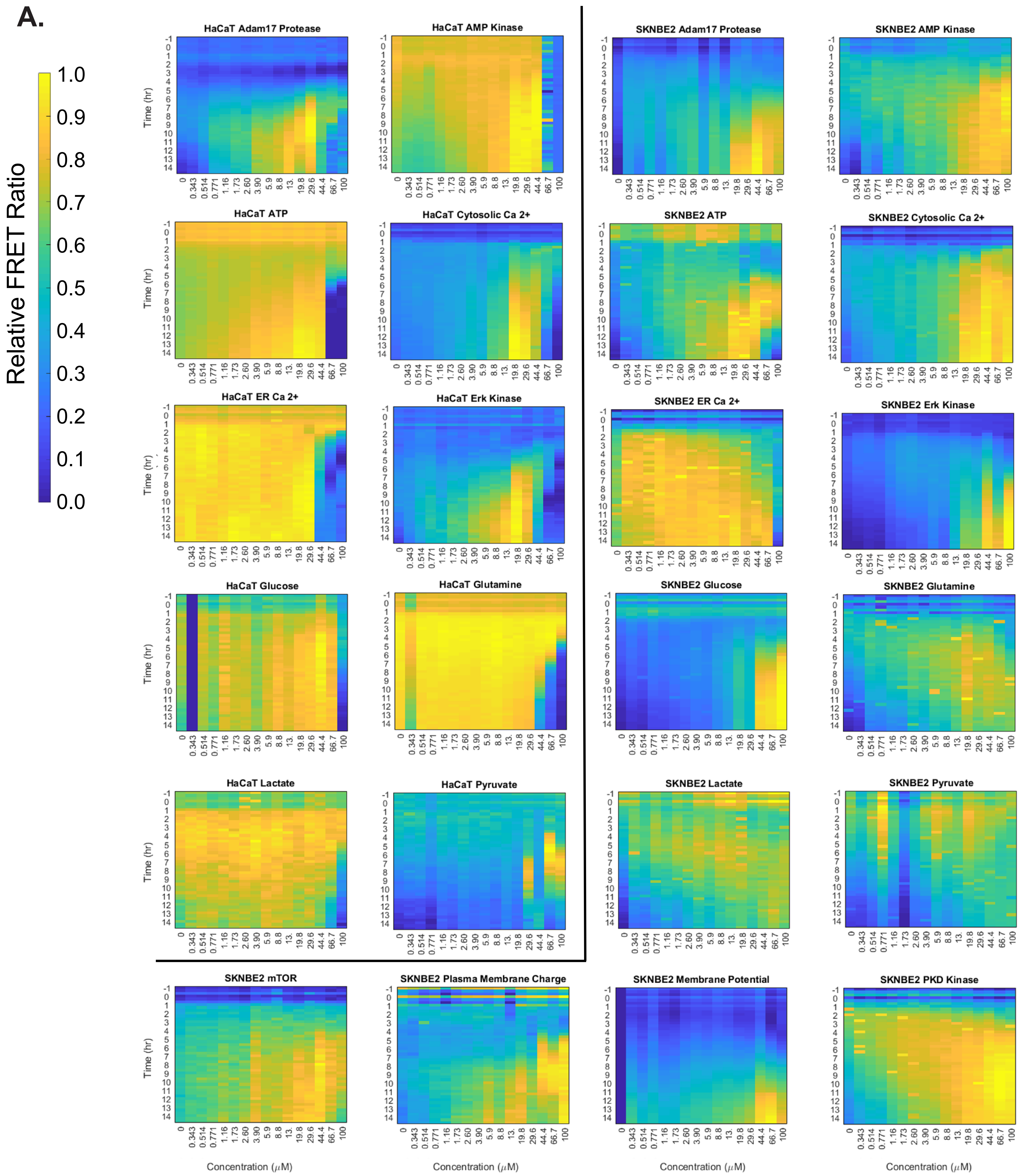

Supplemental Figure 1

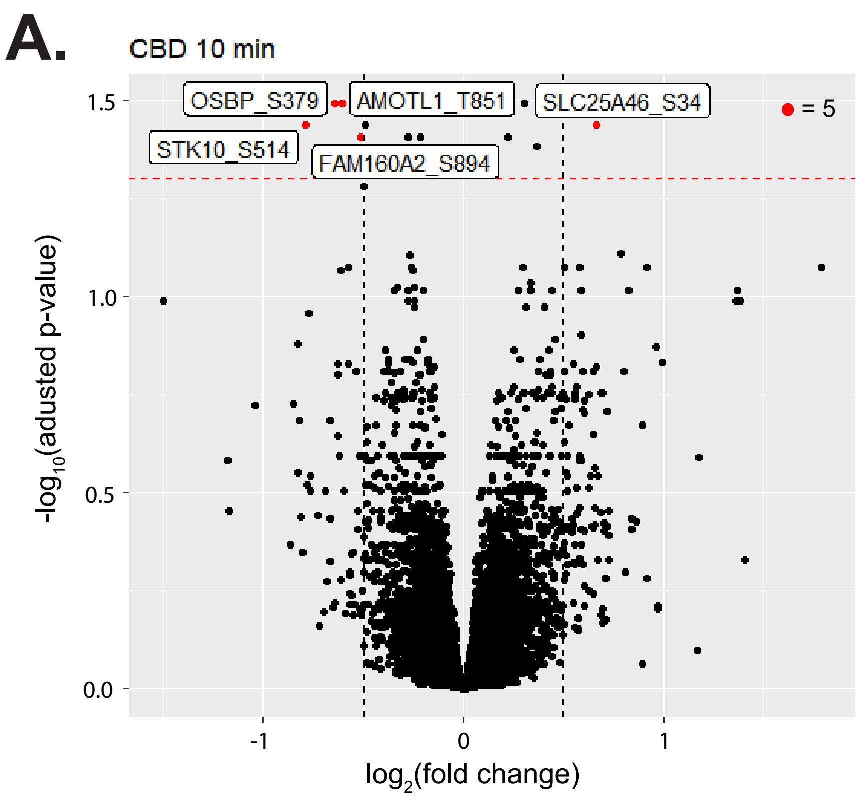

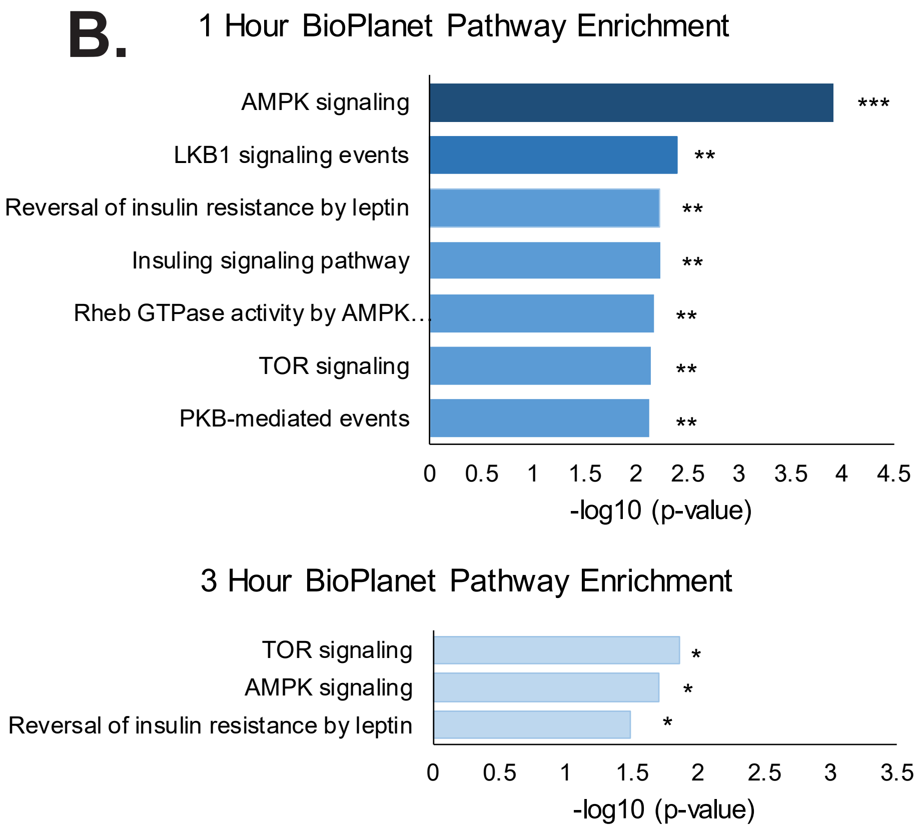

C. **1 Hour post-CBD Treat**

Id	Motif	ZScore	P-Value	Fold Change	Foreground Matches	Foreground Size	Background Matches	Background Size
1	.G..St.S.R.	10.43	0.00E+00	3366.33	2	20	15	504949
2	**..R..s.....**	8.98	0.00E+00	5.26	30	105	43563	802214
3	**..R..s...L.**	7.74	5.11E-15	16.03	8	105	3813	802214
4	.GGS.t.....	7.55	2.11E-14	257.63	2	20	196	504949
5	.GG..t.L...	7.51	2.99E-14	248.74	2	20	203	504949
6	P..S.t..P..	6.93	2.13E-12	159.79	2	20	316	504949
7	...S.t..P..	6.58	2.35E-11	31.93	4	20	3163	504949
8	..R..s..P..	6.55	2.96E-11	14.87	6	105	3082	802214
9	**L.R..s.....**	6.49	4.15E-11	11.94	7	105	4481	802214
10	...S.t.SP..	6.47	4.93E-11	112.96	2	20	447	504949

D. **3 Hour post-CBD Treat**

Id	Motif	ZScore	P-Value	Fold Change	Foreground Matches	Foreground Size	Background Matches	Background Size
1	**..R..s.....**	9.03	0.00E+00	5.31	30	104	43563	802214
2	**..R..s....L**	6.75	7.54E-12	13.17	7	104	4099	802214
3	..R..s..S..	6.34	1.18E-10	11.22	7	104	4813	802214
4	...S.s...L.	5.35	4.51E-08	7.63	7	104	7076	802214
5	R....t..F..	5.15	1.33E-07	40.61	2	30	829	504949
6	.....t.NK..	4.94	3.91E-07	34.88	2	30	965	504949
7	**L....s.....**	4.86	5.88E-07	2.56	25	104	75396	802214
8	L.R..t.....	4.82	7.25E-07	17.01	3	30	2968	504949
9	....Nt.D...	4.79	8.28E-07	31.29	2	30	1076	504949

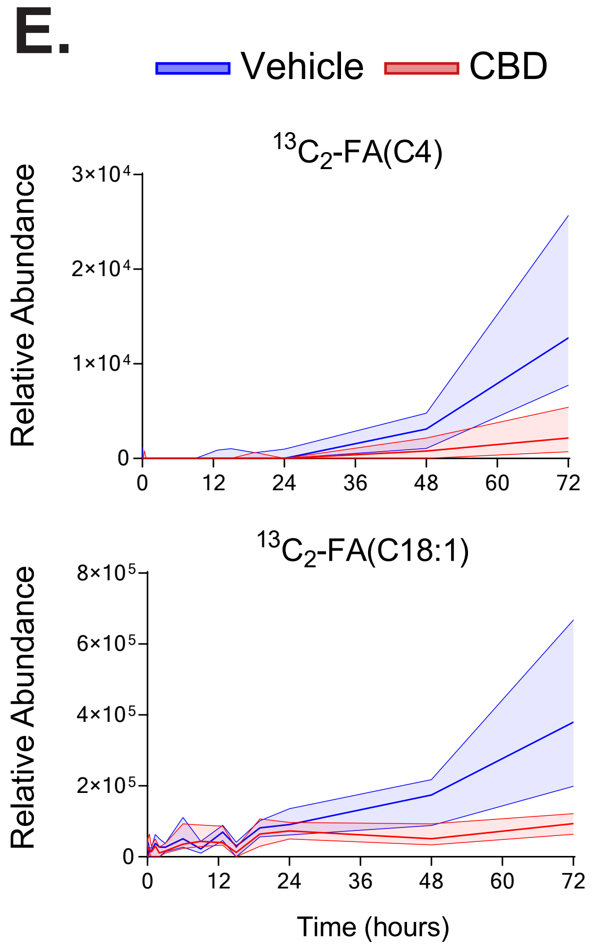

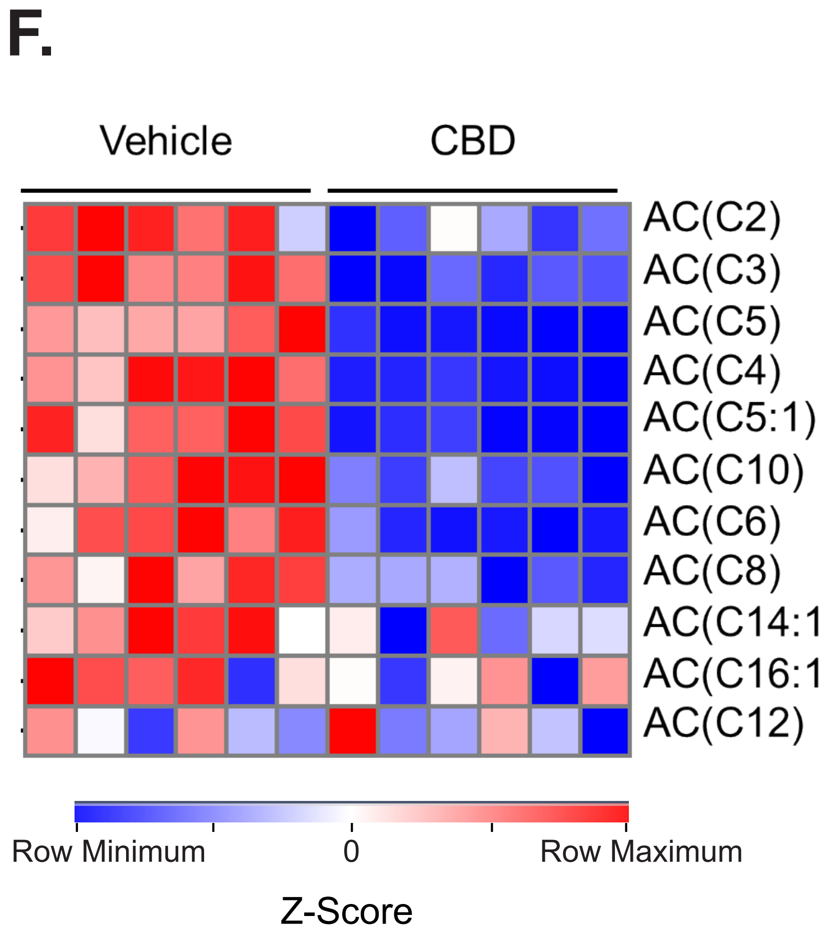

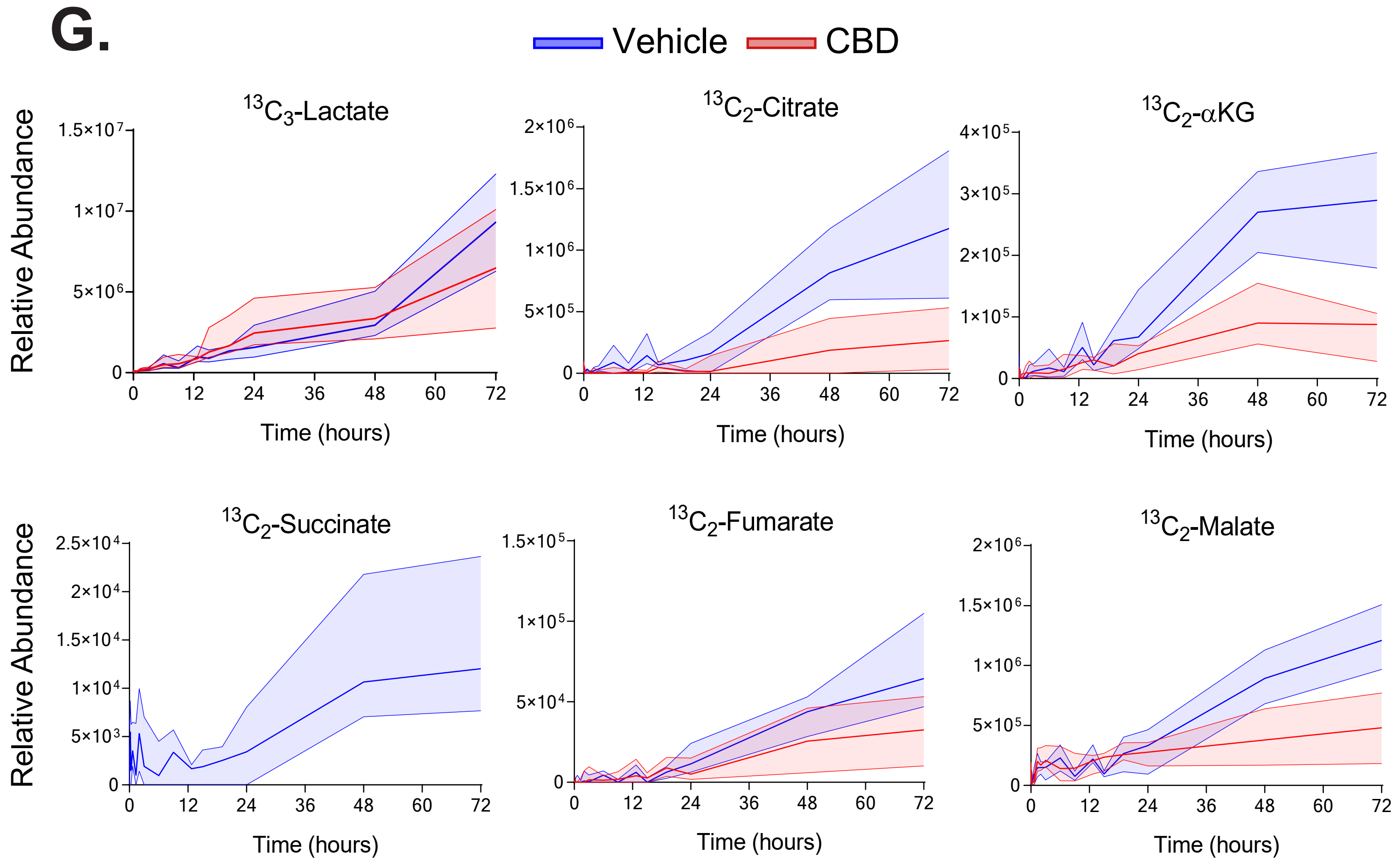

Supplemental Figure 2

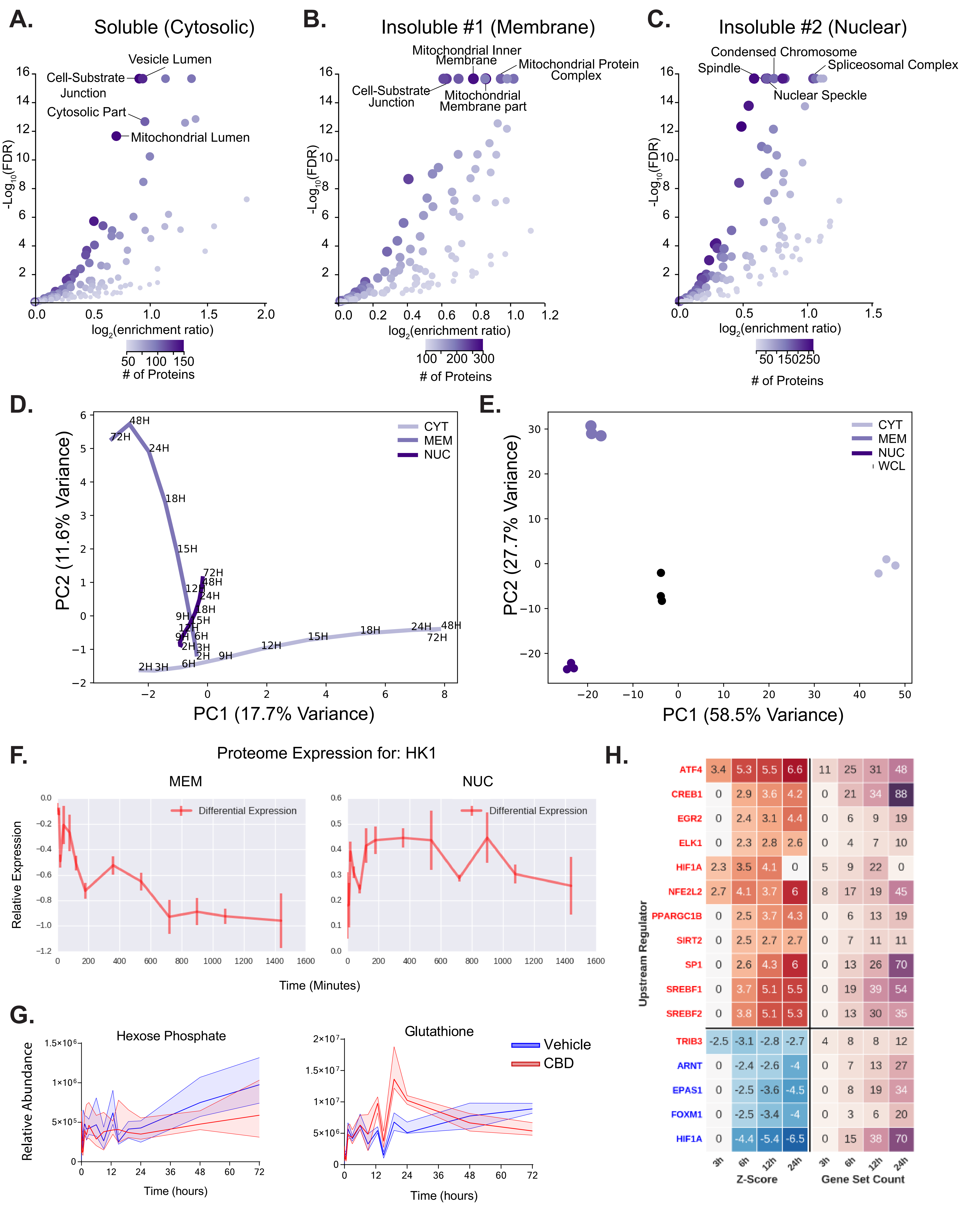

Supplemental Figure 3

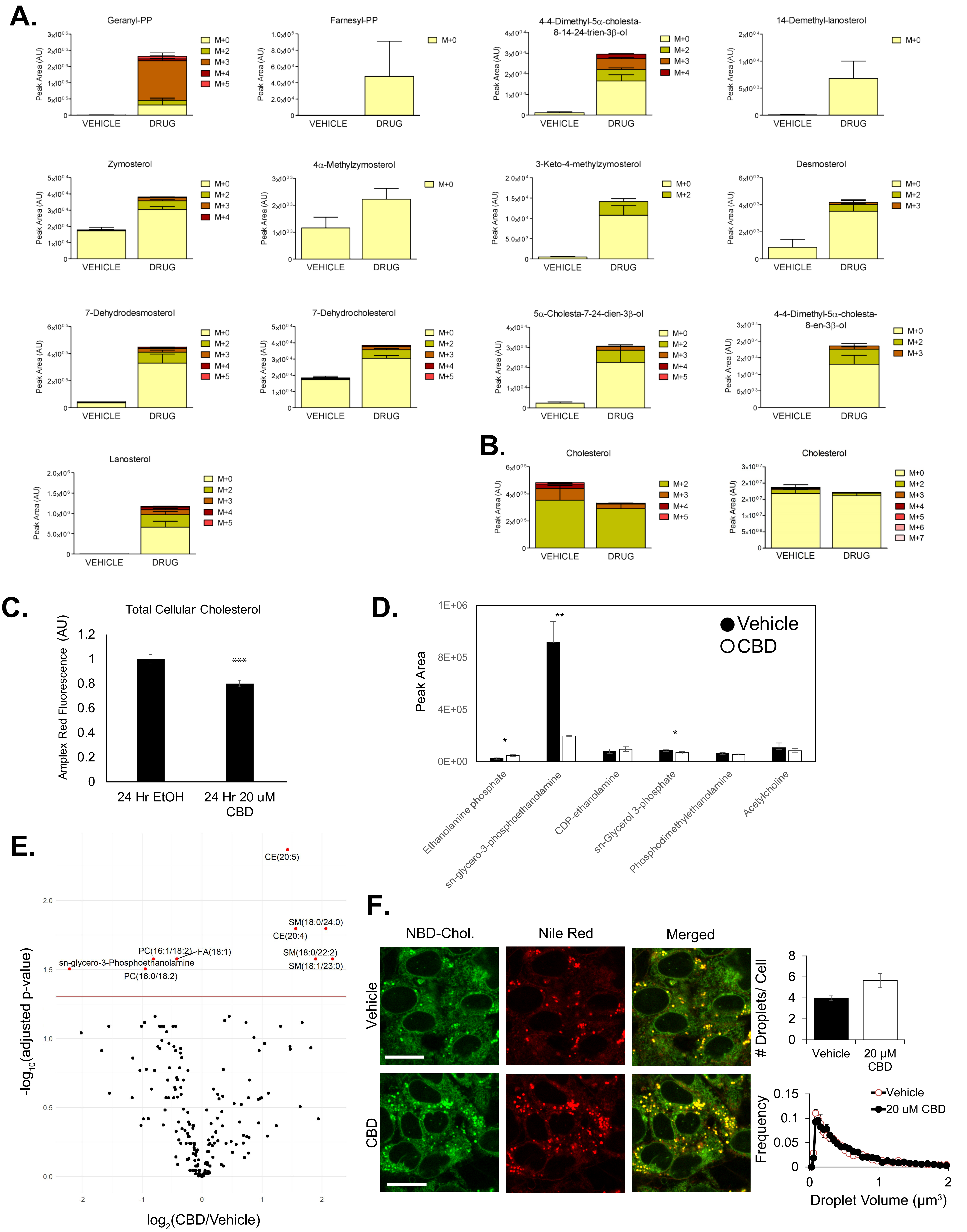

Supplemental Figure 4

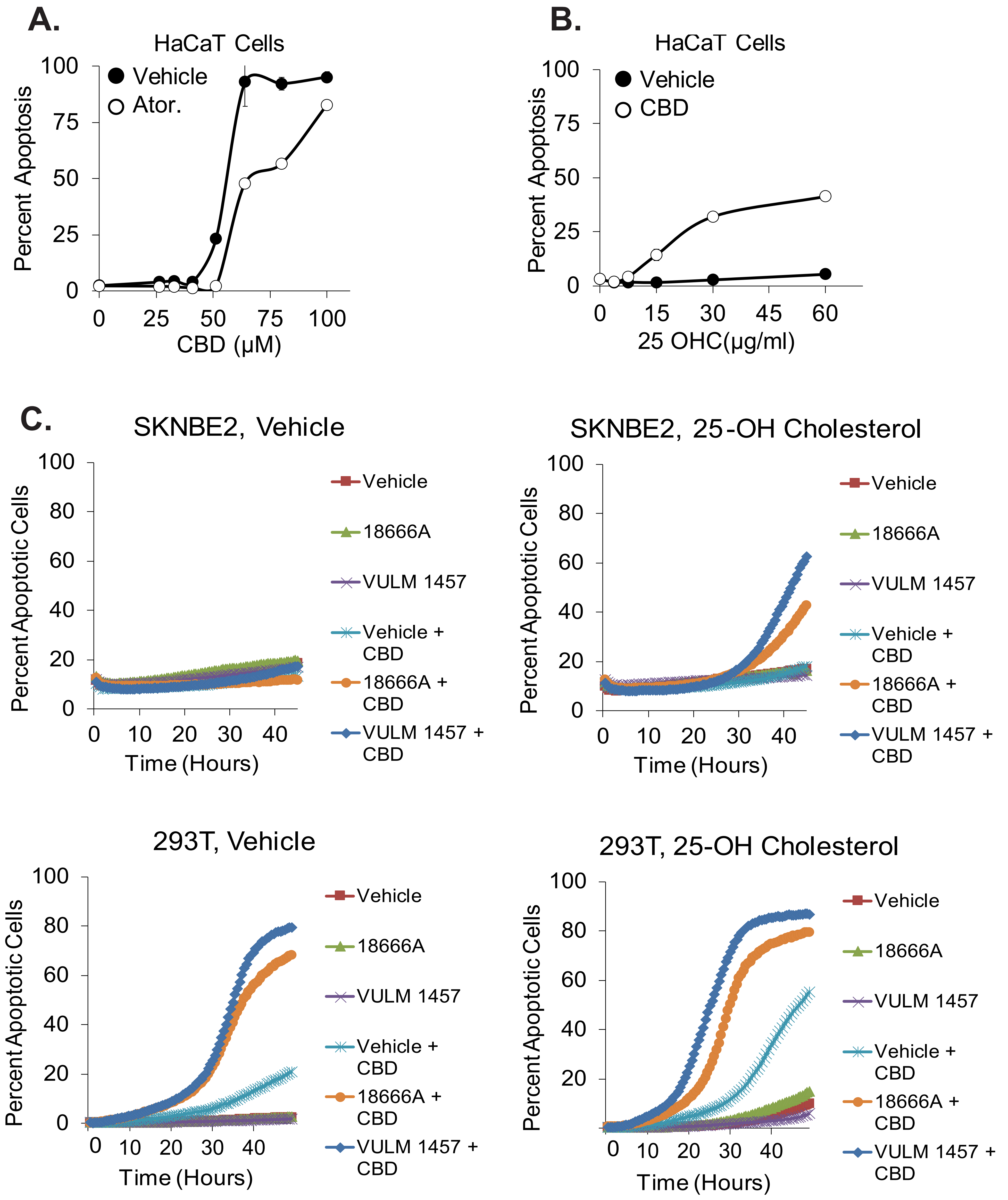

Supplemental Figure 5

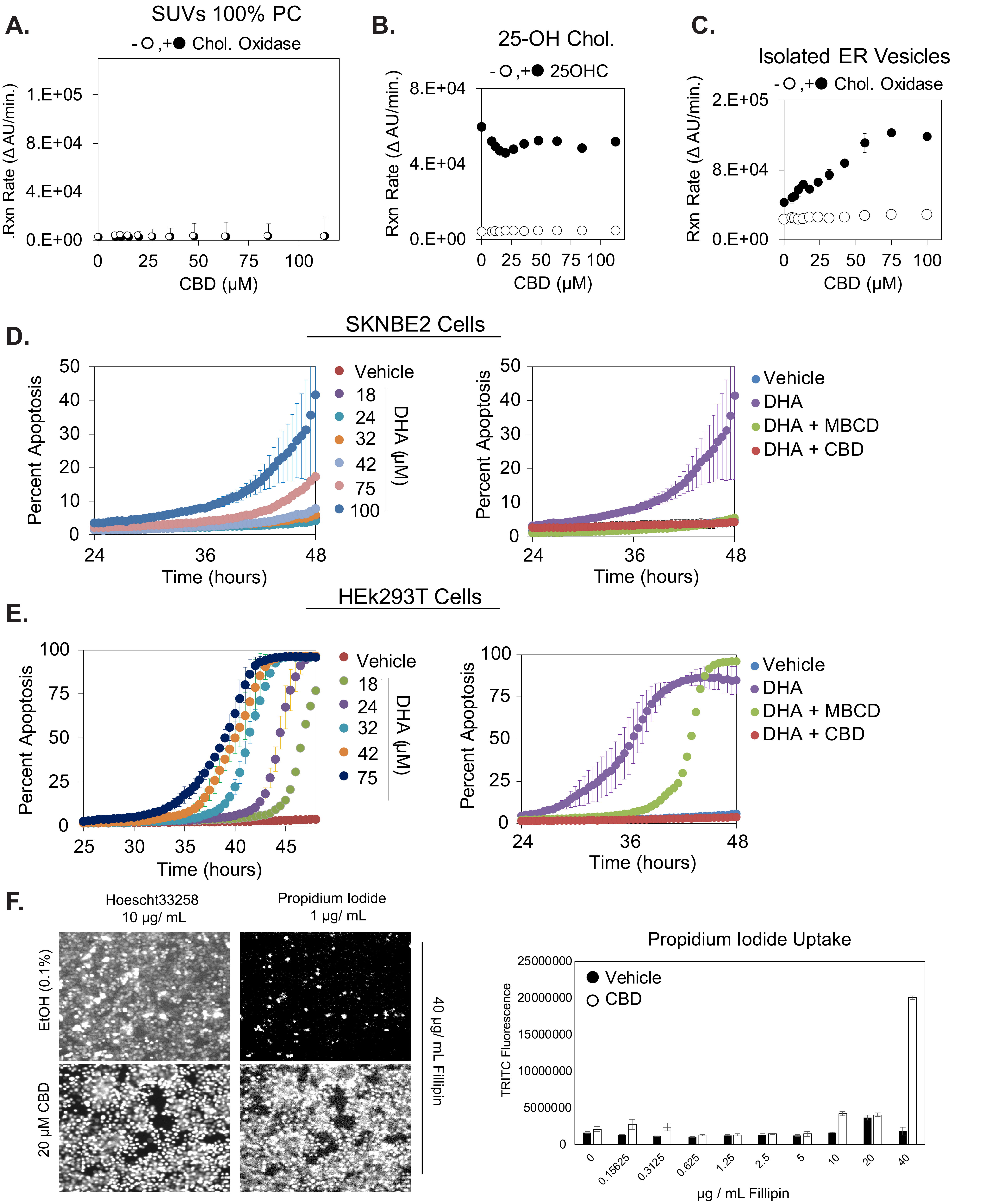

Supplemental Figure 6
